# Maternal TDP43 orchestrates nuclear speckle assembly and zygotic splicing activation during oocyte-to-embryo transition in mice

**DOI:** 10.1093/nar/gkaf1469

**Published:** 2026-01-09

**Authors:** Zuo-Qi Deng, Zhi-Yan Jiang, Yu-Ke Wu, Yun-Wen Wu, Hua Zhang, Lu Chen, Zhi-Yi Li, Li-Ling Liu, Heng- Yu Fan

**Affiliations:** Zhejiang Key Laboratory of Precise Protection and Promotion of Fertility, Assisted Reproduction Unit, Department of Obstetrics and Gynecology, Sir Run Run Shaw Hospital, Life Sciences Institute, Zhejiang University, Hangzhou 310058, China; Zhejiang Key Laboratory of Precise Protection and Promotion of Fertility, Assisted Reproduction Unit, Department of Obstetrics and Gynecology, Sir Run Run Shaw Hospital, Life Sciences Institute, Zhejiang University, Hangzhou 310058, China; Zhejiang Key Laboratory of Precise Protection and Promotion of Fertility, Assisted Reproduction Unit, Department of Obstetrics and Gynecology, Sir Run Run Shaw Hospital, Life Sciences Institute, Zhejiang University, Hangzhou 310058, China; Zhejiang Key Laboratory of Precise Protection and Promotion of Fertility, Assisted Reproduction Unit, Department of Obstetrics and Gynecology, Sir Run Run Shaw Hospital, Life Sciences Institute, Zhejiang University, Hangzhou 310058, China; Zhejiang Key Laboratory of Precise Protection and Promotion of Fertility, Assisted Reproduction Unit, Department of Obstetrics and Gynecology, Sir Run Run Shaw Hospital, Life Sciences Institute, Zhejiang University, Hangzhou 310058, China; Zhejiang Key Laboratory of Precise Protection and Promotion of Fertility, Assisted Reproduction Unit, Department of Obstetrics and Gynecology, Sir Run Run Shaw Hospital, Life Sciences Institute, Zhejiang University, Hangzhou 310058, China; State Key Laboratory of Female Fertility Promotion, Center for Reproductive Medicine, Department of Obstetrics and Gynecology, Peking University Third Hospital, Beijing 100191, China; Department of Computer Science and Engineering, College of Engineering, The Ohio State University-Columbus, Columbus, OH 43210, United States; Department of Reproductive Medicine and Genetics Center, The People’s Hospital of Guangxi Zhuang Autonomous Region, Nanning 530021, Guangxi, China; Zhejiang Key Laboratory of Precise Protection and Promotion of Fertility, Assisted Reproduction Unit, Department of Obstetrics and Gynecology, Sir Run Run Shaw Hospital, Life Sciences Institute, Zhejiang University, Hangzhou 310058, China; Department of Reproductive Medicine and Genetics Center, The People’s Hospital of Guangxi Zhuang Autonomous Region, Nanning 530021, Guangxi, China

## Abstract

Zygotic splicing activation (ZSA) is a crucial process of mRNA post-transcriptional regulation during maternal-to-zygotic transition, ensuring normal embryonic development. However, the key factors and mechanisms underlying ZSA regulation remain unclear. We observed that the nuclear speckle (NS), a key splicing region, was newly established at the 2-cell stage in mice and was consistent with the ZSA period. Moreover, NS and TAR DNA-binding protein-43 (TDP43) always exhibited a partially adjacent and mutually exclusive localization relationship. TDP43 performed its function through liquid–liquid phase separation. The condensation-deficient state of TDP43 was the active form involved in NS regulation in 2-cell embryos. Additionally, TDP43 could also interact with both transcribed RNAs associated with NS and directly with NS proteins. Maternal TDP43 deficiency led to the failure of NS assembly in 2-cell embryos, resulting in the inability to skip exons during transcript splicing. In contrast, ectopic expression of TDP43 in zygotes led to abnormal enlargement of the NS, resulting in excessive skipping of transcript exons. Both bidirectional ZSA disorders led to 2-cell arrest during early embryogenesis. ZSA defects caused by TDP43 deficiency also impaired cell totipotency-pluripotency conversion. In this study, we identified an NS upstream regulatory factor, TDP43, which helps maintain the balance of ZSA, providing a new perspective on the post-transcriptional regulation of early embryos.

## Graphical Abstract

**Graphical Abstract:**
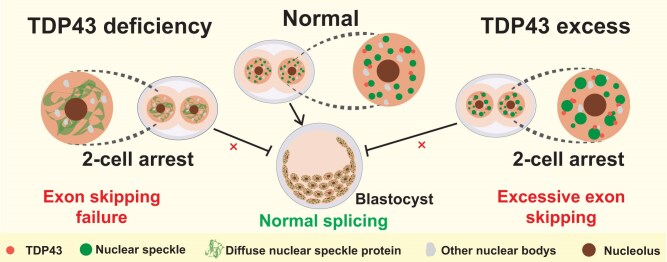

## Introduction

Fertilization is the origin of life and is accompanied by the reprogramming of transcripts, including the degradation of maternal mRNA and the initial production of zygotic mRNA. This process is defined as maternal-to-zygotic transition (MZT) [1]. Maternal mRNAs are fundamental for oocyte development, and their clearance determines the developmental competence of fertilized eggs. Defects in maternal factor-mediated mRNA decay (M-decay) or zygotic factor-mediated mRNA decay (Z-decay) can lead to arrest in embryonic development [2–7]. Zygotic genome activation (ZGA), a highly conserved physiological process typical of vertebrates, marks a switch in genetic control from the maternal to the embryonic genome [1, 8]. It was recently reported that the transcription factors that underlie mammalian ZGA, including OBOX1, NR5A2, and TPRXs [8–10], are required for embryonic development. Although considerable progress has been made in understanding transcriptional activation, the post-transcriptional regulatory mechanisms of ZGA products require further investigation.

As an important mechanism of post-transcriptional genome regulation, the process of alternative splicing (AS) is evolutionarily conserved in various organisms. Therefore, expanding the functional and regulatory abilities of eukaryotic genomes to produce diverse transcripts and protein isoforms is essential. Different cell and tissue types exhibit different AS patterns [11], and defects in cell differentiation, cell fate decisions, and development are observed when these patterns are altered [12–14]. Studies have revealed that the AS patterns of human and mouse embryos share similarities with many alternative splicing events (ASEs) occurring during ZGA, a process known as zygotic splicing activation (ZSA) [15]. ZSA depends on the MZT process; therefore, either failure of ZGA or interference with maternal splicing factors leads to ZSA disorders [15]. ZSA is essential for early embryonic development, including transcriptional regulation and control of totipotency-pluripotency conversion. However, certain key questions—including how ZSA is initiated, which key factors regulate ZSA, and how related factors are assembled—remain unanswered.

The cell nucleus is a highly compartmentalized structure with various nuclear bodies, including the nuclear speckle (NS), transcription-related body, Cajal body, paraspeckle, and nucleolus, which compartmentalize the nuclear space to perform various biochemical functions [16]. NSs are widely regarded as important splicing factors that mediate various physiological activities. Impairment of the NSs leads to disordered AS, resulting in defects in cell development and the development of cancer and other diseases [17]. However, most current research on NS is based on somatic cells. The dynamic changes in NSs in pre-implantation embryos lack a detailed description, and whether ZSA is dependent on NS remains unclear.

The NS is a complex composed of heterogeneous components, including multiple RNAs and proteins. Certain important splicing factors, such as serine/arginine-rich proteins (SR proteins), have been observed in the NS, a family of RNA-binding proteins (RBPs) named after their intrinsically disordered serine and arginine residues. SRSF2 (SC35), an SR protein, is localized to the central region of the NS and is necessary for NS splicing. Additionally, some spliceosomes are localized at the periphery of the NS [18, 19]. ZC3H14, an evolutionarily conserved Cys3His zinc finger protein, co-localizes with SRSF2 and plays a role in mRNA processing, suggesting that it is also a component of the NS [20]. Moreover, Dai *et al.* reported that non-membrane-bound compartments, known as nuclear poly(A) domains (NPADs), are mediated by PABPN1, which co-localizes with the NS structure [21], in developing mouse oocytes. This indicates that NPAD is a unique NS in oocytes. Although multiple proteins and RNAs have been identified as components of the NS, the upstream regulatory elements facilitating NS assembly remain largely unknown.

TAR DNA-binding protein-43 (TDP43) is an abundant and ubiquitously expressed RBP that regulates RNA metabolism. Numerous studies have demonstrated that TDP43 affects the splicing process [22–24]. The disruption of TDP43 leads to splicing disorders that are harmful to normal physiological activities. However, the precise role of TDP43 in splicing remains unclear, and further investigation is required to determine whether TDP43-mediated splicing regulation is related to NSs.

TDP43 is a classic liquid–liquid phase separation (LLPS) molecule. Studies have shown that the phase separation behavior of TDP43 is significantly affected by its C-terminal low-complexity region. The disordered nature of this region enables TDP43 to form droplet-shaped membrane-less organelles in cells. The distribution and dynamic changes in these droplet-shaped structures in cells are crucial for regulating cellular functions. Abnormally aggregated TDP43 in the cytoplasm is closely associated with various neurodegenerative diseases [23, 25, 26]. TDP43 aggregates form biological condensates such as stress granules within cells in response to cell damage [27]. The function of TDP43 under abnormal pathological or stress conditions has been extensively studied. However, it remains unclear how condensates formed by TDP43 are distributed and dynamically change under normal physiological conditions, such as during early embryonic development, and whether these condensates are spatially or functionally related to NS condensates.

In this study, we revealed that TDP43 undergoes LLPS under normal physiological conditions and provided detailed descriptions of the dynamic changes between NS and TDP43 in mouse oocytes and pre-implantation embryos. Using knockout mouse models and overexpression systems, we revealed the importance of TDP43 in maintaining NS balance. The regulatory effect of TDP43 on NSs is mainly mediated by its RNA-binding ability and the balance between the condensation-deficient and condensation-preserving states. The results of this study revealed that, in addition to its participation in splicing, TDP43 also regulates ZSA through NSs and mediates mouse embryonic development.

## Materials and methods

### Mice

The mice used in this study possessed either mixed or C57BL/6 genetic backgrounds. ICR WT mice were purchased from Hangzhou Medical College. All animal experiments were conducted in accordance with the guidelines and regulations of Zhejiang University, and the experimental protocol (ZJU20210252) was approved by the Zhejiang University Institutional Animal Care and Research Committee.

### Oocyte collection, superovulation, and *in vitro* culture

Fully grown oocytes (FGOs) were collected 48 h after female mice were injected with 5 IU PMSG, and GOs were collected from 14-day-old female mice. Metaphase II (MII) oocytes were deprived of oocyte/cumulus masses, digested with 0.3% hyaluronidase (Sigma–Aldrich) after 48 h, and injected with 5 IU PMSG, and with 5 IU hCG a further 16 h later. All those oocytes were cultured in M2 medium (M7167; Sigma–Aldrich) covered with mineral oil (M5310; Sigma–Aldrich) under 5% CO_2_ at 37°C.

### Fertilization and embryo culture

To obtain naturally fertilized eggs, super-ovulated female mice were mated with 10–12 week-old wild-type (WT) male mice overnight. The cumulus–oocyte complexes (COC) of successfully mated mice with vaginal plugs were digested using 0.3% hyaluronidase. The zygotes were cultured in KSOM medium (M1435; Nanjing AIBI Bio-Technology Co., Ltd) covered with mineral oil at 37°C in 5% CO_2_. To synchronize zygotic development, we adopted previously reported methods to obtain *in vitro* fertilized embryos [28, 29]. Human tubal fluid (HTF) medium (M1135; Nanjing AIBI Bio-Technology Co., Ltd) was used for sperm capacitation and fertilization. Oocytes and sperm were cultured in HTF medium at 37°C in 5% CO_2_ for 4–6 h to achieve full fertilization. We considered the time when sperm were added to the COC as *in vitro* fertilization (IVF) 0 h. *In vitro* fertilized eggs were washed at IVF 4.5 h in M2 medium and transferred to KSOM medium covered with mineral oil for continuous culture at 37°C in 5% CO_2_.

### Live cell imaging

GOs microinjected with *mCherry-Pabpn1* and *Flag-Egfp-Tdp43* mRNA were cultured in the M2 medium. A solid medium derived from 1% low-density agar powder (BBI A600015-0025) dissolved in 1× phosphate buffered saline (PBS) was used to fix the samples. We heated the solid culture medium in a 40°C metal bath and poured it into a glass Petri dish. After 4–6 h of mRNA expression, we transferred GOs to the medium and waited at room temperature for the GOs to sink to the bottom and for the culture medium to solidify. Live cell imaging was performed using Olympus SpinSR in 5% CO_2_ at 37°C.

### Cell culture and transfection

Human embryonic kidney (HEK) 293T, HeLa, ES-2, mOSE, and SKOV3 cells were all cultured in DMEM supplemented with 10% fetal bovine serum (FBS) and 1% penicillin/streptomycin at 37°C in 5% CO_2._ GCs were isolated from female mice at 24 h after PMSG injection and cultured in DMEM supplemented with 5% FBS and 1% penicillin/streptomycin at 37°C in 5% CO_2_. The cells were transfected with plasmids using Lipofectamine 2000 (Invitrogen) in Opti-MEM (Invitrogen). The transfection time was at least 24 h before subsequent immunofluorescence or other experiments.

### Immunofluorescence

Oocytes, embryos, and other cells were fixed with 4% PFA in 1× PBS for 30 min at room temperature, permeabilized in PBS with 0.3% Triton X-100 for 30 min at room temperature, blocked in washing buffer with 1% BSA at room temperature, and then incubated overnight at 4°C with primary antibodies diluted in blocking solution. Next, the samples were washed thrice for 5 min in washing buffer that was 1× PBS with 0.01% Triton X-100/0.16% Tween-20 and incubated with secondary antibodies diluted 1:200 in blocking solution for 30 min at room temperature. Finally, the samples were washed thrice with washing buffer for 5 min each and mounted in a glass-bottom dish. Imaging of the samples after immunofluorescence was performed using a Zeiss LSM880 or LSM900 confocal microscope. ImageJ software was used to process the images and quantify the fluorescence signals. All antibodies used are listed in Supplementary Table S1.

### EU incorporation assay

A 5-ethynyl-uridine (EU) incorporation assay was performed to detect the RNA transcriptional activity. The 2-cell embryos were cultured in KSOM supplemented with 1 mM EU for 1 h and fixed in 4% PFA in 1× PBS for 30 min at room temperature. The Cell-Light^TM^ Apollo 567 Stain Kit was used to display fluorescent signals, according to the manufacturer’s protocol.

### Detection of protein synthesis

l-homopropargylglycine (HPG) can be incorporated into nascent proteins and reflects the overall protein translation status through fluorescent signals. Embryos were incubated with 20 μm HPG diluted in KSOM medium for 30 min using the Click-iT protein synthesis assay kit. The embryos were fixed, and fluorescent signals were visualized.

### Plasmid linearization and *in vitro* transcription and mRNA microinjection

Plasmid DNA was linearized using single-enzyme digestion for *in vitro* transcription. Linearized DNA was dissolved in nuclease-free water using the DNA Gel Recovery Kit (Axygen). The corresponding transcription kit was selected according to the different promoters in the plasmids for *in vitro* transcription using the mMESSAGE mMACHINE SP6 Kit (Invitrogen, AM1340) for plasmids with the SP6 promoter and the mMESSAGE mMACHINE T7 Kit (Invitrogen, AM1344) for plasmids containing the T7 promoter. Pre-mRNA was *in vitro*-polyadenylated using the Poly(A) Tailing Kit (Invitrogen, AM1350) according to the manufacturer’s protocol. Lithium chloride was used to precipitate the mRNA overnight at −20°C. Finally, the mRNA pellet was washed thrice with 75% ethanol for 5 min and dissolved in nuclease-free water to obtain clean mRNA. All mRNAs were diluted to 500–800 ng/µl and stored at −80°C for microinjection.

### Western blotting analysis

Oocytes and embryos were lysed with 20 µl of 2× sodium dodecyl sulfate (SDS) and boiled at 95°C for 10 min to ensure complete protein denaturation. 293T cells in 12-well plates were lysed with 200 µl of 2 × SDS/well and heated at 95°C for 15 min. Lysates were resolved on 8%–10% sodium dodecyl sulfate–polyacrylamide gels and transferred to PVDF membranes activated with methanol. The blots were blocked with 5% milk and then incubated with primary antibodies overnight at 4°C. After washing three times in 1× TBST for 5 min each wash, the blots were incubated with secondary antibodies for 30 min at room temperature, washed again, and finally developed with a chemiluminescent substrate. The primary antibodies and their dilutions are listed in Supplementary Table S1.

### Immunoprecipitation

The cells were lysed in lysis buffer (50 mM Tris–HCl, pH 7.5, 150 mM NaCl, 10% glycerol, and 0.5% NP-40; protease and phosphatase inhibitors were added before use) for 20 min at 4°C after transfection of the corresponding plasmids for 48 h. After centrifugation at 12 000 × *g* for 20 min, a portion of the supernatant was used as the input, and the remaining supernatant was collected and subjected to immunoprecipitation with corresponding beads for 4 h at 4°C. The beads were then washed three times with lysis buffer, and SDS buffer was added. Finally, the input and eluates were heated at 95°C for 10 min to denature the protein and then used for western blot analysis. Endogenous immunoprecipitation was performed using protein A beads coupled with TDP-43 antibody or rabbit IgG. The remaining steps were the same as those described above.

### RNA isolation and qRT-PCR

In total, 10 oocytes or embryos were washed in 0.2% BSA in 1× PBS and then lysed in 2 µl 0.2% Triton X-100 with 2 IU/µl RNase inhibitor. Total RNA was reverse transcribed using PrimeScript II reverse transcriptase (Takara, 6210A) and random primers (Takara, 3801). The PCR products were diluted two- to three-fold and subsequently used for semi-quantitative RT-PCR with Power SYBR Green PCR Master Mix (Applied Biosystems, Life Technologies). Relative mRNA levels were calculated by normalizing to the levels of endogenous *Gapdh* mRNA (internal control) using Microsoft Excel. All primer sequences used in this study are listed in Supplementary Table S2.

### Library construction of poly(A) RNA sequencing

Ten embryos per sample were washed in 0.2% BSA in 1× PBS and collected for lysis in 4.2 µl lysis buffer [0.2% Triton X-100, RNase inhibitor, deoxyribonucleotide triphosphate (dNTPs), oligo-dT primers, and 1:1000 ERCC spike-in]. The samples were reverse transcribed and amplified using the Smart-seq2 method as previously described [30]. Sequencing libraries were constructed from 500 pg of the amplified complementary DNA (cDNA) using the TruePrep DNA Library Prep Kit V2 for Illumina (Vazyme, TD503). Barcoded libraries were pooled and sequenced on the HiSeq X Ten platform with 150-bp paired-end reads. RNA-seq quality control data are provided in Supplementary Table S3.

### Transcriptome analyses

Raw data were filtered using low-quality reads and an adapter using TrimGalore. Clean reads were then mapped to the mouse genome by mm10 using the STAR software and further normalized to the ERCC spike-in. The DESeq2 R package was used for DEGs analysis. All DEGs were within the range of adjusted *P*-value < 0.05 and fold change (FC) of *Tdp43^♀–/♂+^*/*Tdp43^♀+/♂+^ ≥ *2 or ≤ 0.5. Counts and fragments per kilobase per million (FPKMs) normalized to ERCC were calculated to estimate gene expression levels. Feature counts of Smart-seq-derived transcripts are provided in Supplementary Tables S4 and S5.

### Analysis of various alternative splicing events

rMATS software was used to analyze five different ASEs: SE, A5SS, A3SS, mutually exclusive exons (MXEs), and intron retention (RI). All ASEs were within the range of false discovery rate (FDR) < 0.05, and the Inc Level Difference was ≥ 0.1 or ≤ −0.1.

### Library construction and analysis of ribonucleoprotein immunoprecipitation RNA sequencing

The ribonucleoprotein immunoprecipitation (RIP) assay was performed as previously described [31]. A lysis buffer [50 mM Tris–HCl (pH 7.4), 1% Triton X‐100, 150 mM NaCl, 5 mM EDTA, protease inhibitor cocktail, and RNase inhibitor] was used to lyse 400 2-cell embryos from each sample. A total of 90% cell lysate supernatant was used for immunoprecipitation with agarose beads conjugated with IgG or FLAG antibodies, while the residual cell lysate supernatant was used as the “input.” After incubation at 4°C for 4 h, the bead-bound RNAs were collected and reverse transcribed. RNA was amplified for library construction using the Smart-seq2 method as described previously [30]. RIP-seq quality control data are provided in Supplementary Table S3. FPKM values of RIP-seq-derived transcripts are presented in Supplementary Table S6.

### Statistical analysis

All statistical data presented in this study are means ± SEM drawn with GraphPad Prism 8 software. All statistical experiments were performed at least three times. A two-tailed unpaired Student’s *t*-test was used to calculate significant differences between the two groups. Statistically significant values of *P* < 0.05, *P* < 0.01, *P* < 0.001, and *P* < 0.0001 obtained using the two-tailed Student’s *t*-test are indicated by asterisks (*), (**), (***), and (****), respectively. “n.s.” indicates non-significant.

## Results

### TDP43 undergoes a phase transition process under normal physiological conditions

**Figure 1.**
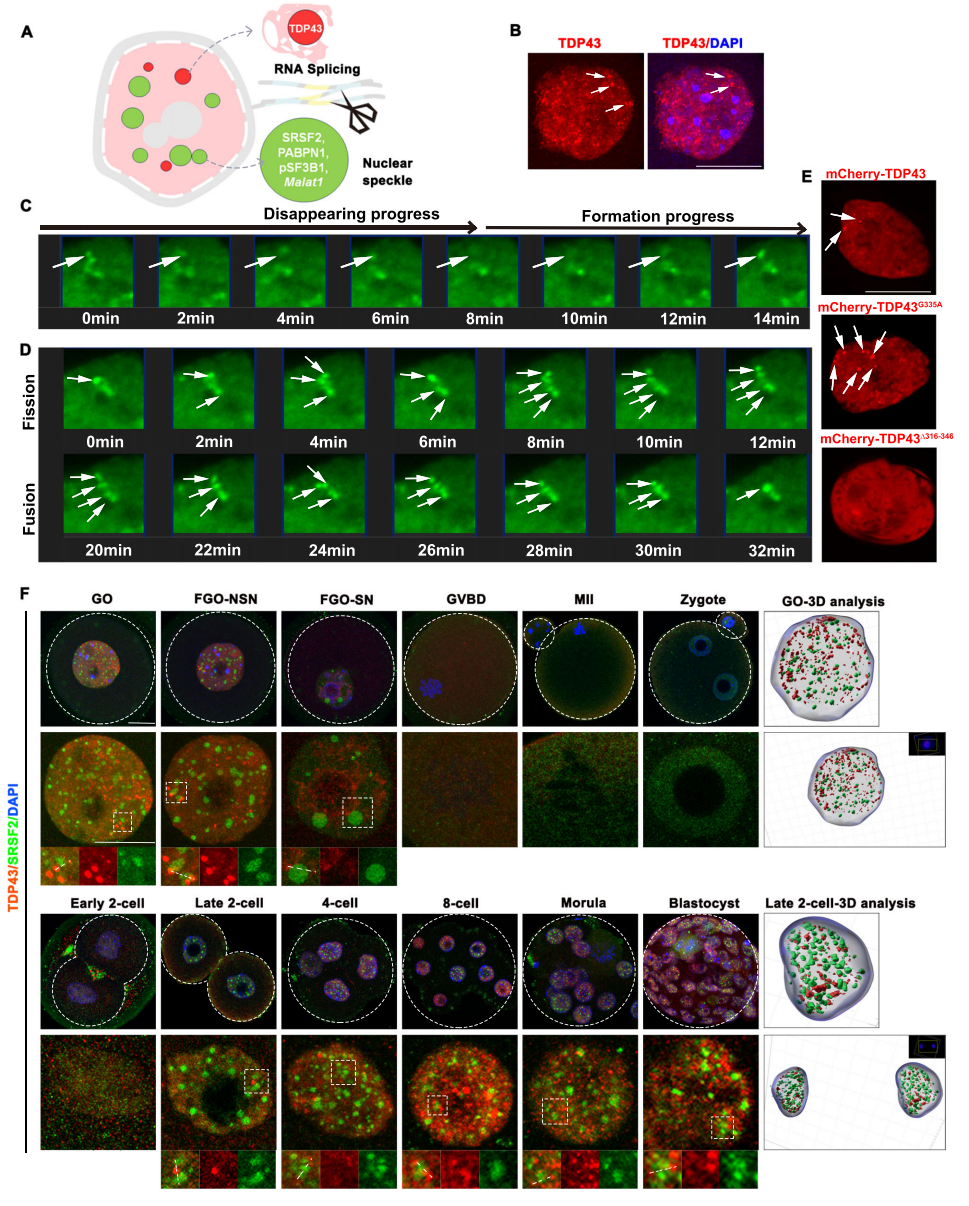
Dynamic changes of TDP43 and SRSF2 during mouse oocyte to pre-implantation embryo development. (**A**) A diagram indicating the morphology of NSs and TDP43 in somatic cells and the important components of NSs. (**B**) The distribution of endogenous TDP43 in the nucleus of GOs. Scale bar = 20 μm. (**C,D**) Time-lapse images of growing oocyte nuclei expressing GFP-TDP43. A dynamic progress of droplet-like TDP43 occurring in the growing oocyte nucleus, which is indicated using a arrow in the figure. (**E**) The distribution of TDP43 and its mutants in the nuclei of GOs after injection. (**F**) Immunofluorescence confocal microscopy images indicating the dynamic patterns of TDP43 and SRSF2 during mouse oocyte maturation and pre-implantation embryo development. The enlarged signal diagram is presented below. The 3D model diagram of GO and 2-cell analyzed using Imaris is presented on the right. Scale bar = 20 μm.

Although TDP43 can form droplet-like condensates both *in vivo* and *in vitro, in vivo* studies have primarily focused on neural cells. However, it is unclear whether TDP43 forms condensate structures in germ cells. The formation of droplet-like phase transition structures depends on RNA concentration. Distribution and dynamic changes in splicing regulatory factors, including TDP43 and NS condensates, were investigated (Fig. 1A). Endogenous TDP43 protein was localized in the nucleus in growing oocytes (GOs) with high transcriptional activity and had apparent dot-like structures (Fig. 1B). We performed live cell imaging by microinjecting *Gfp*-*Tdp43* mRNA into GOs to determine whether TDP43 condensates in oocytes have phase separation properties, which are characterized by internal dynamic reorganization and rapid external exchange [32]. TDP43 condensates gradually disappeared and then reformed (Fig. 1C). TDP43 condensates split from a single large spot into multiple small spots and then reversibly fused into a large spot (Fig. 1D). We proposed that the time-coordinated fusion and division processes of TDP43 are closely associated with its functions. Furthermore, we verified the phase transition properties by interfering with key sites at the C-terminus of TDP43. TDP43 formed very few condensates under normal circumstances, the mutant TDP43^G335A^ had abnormally more condensates, and TDP43^Δ316–346^ barely formed condensates (Fig. 1E). This was consistent with the observation in HEK 293T cells [33]. These results indicated that TDP43 undergoes LLPS in female germ cells.

### TDP43 is partially positioned adjacent to NS in oocytes and somatic cells

Considering that the accumulation of large amounts of mRNAs in oocytes during growth plays an important role in the MZT, we speculated that NSs, the classic RNA processing sites, exhibit a series of dynamic changes that may be a manifestation of their function. SRSF2-labeled NSs and TDP43 were analyzed across multiple cell models, including 293T cells, HeLa cells, human ovarian cancer lines (ES-2, SKOV3), mouse granulosa cells (GCs), and immortalized mouse ovarian epithelial cells (mOSE), to investigate the expression and dynamics of the two bio-condensates (NS and TDP43) (Supplementary Fig. S1A). Although droplet-like TDP43 is typically smaller and more diffused in the nucleoplasm than NSs, a proximal positioning relationship between these two nuclear bodies always exists in somatic cells, except during dividing cells (Supplementary Fig. S1A).

NSs gradually enlarged during oocyte development, and their condensates peaked in FGOs characterized by surrounded nucleolus (SN). Additionally, the number of NSs gradually decreased from GOs to FGO-SNs (Fig. 1F). Once meiosis resumed, the NSs diffused and reappeared as condensates until the late 2-cell stage after fertilization. NS condensates were maintained during various stages of early embryonic development after the 2-cell stage, except in cells undergoing mitosis. Additionally, droplet-like TDP43 is abundant in oocytes and early embryos with a high degree of differentiation and is located adjacent to NSs (Fig. 1F and Supplementary Fig. S1B and C). These results indicated that the adjacent positioning relationship between NSs and TDP43 is conserved in multiple cell types, suggesting functional connections.

Notably, we determined that TDP43 and NSs exhibited a mutually exclusive localization relationship in both oocytes and 2-cells by overexpressing fluorescently labeled proteins (Supplementary Fig. S1D and E). This mutually exclusive localization was mainly reflected in the condensation-deficient state of TDP43, suggesting that the condensation-deficient and condensation-preserving states of TDP43 produced during the phase transition may have different functions. TDP43 was consistently expressed in both mouse oocytes and preimplantation embryos (Supplementary Fig. S1F and G). Furthermore, TDP43 has a unique localization distribution during the 2-cell period; that is, TDP43 is mainly in a condensation-deficient state in the nucleus. This differed from the more condensation-preserving state in the late embryo. Considering that the 2-cell stage is a critical period for the massive reconstruction of NSs after fertilization, the unique localization of TDP43 at this stage may be related to the formation of NSs.

### TDP43 is essential for early embryo development

**Figure 2.**
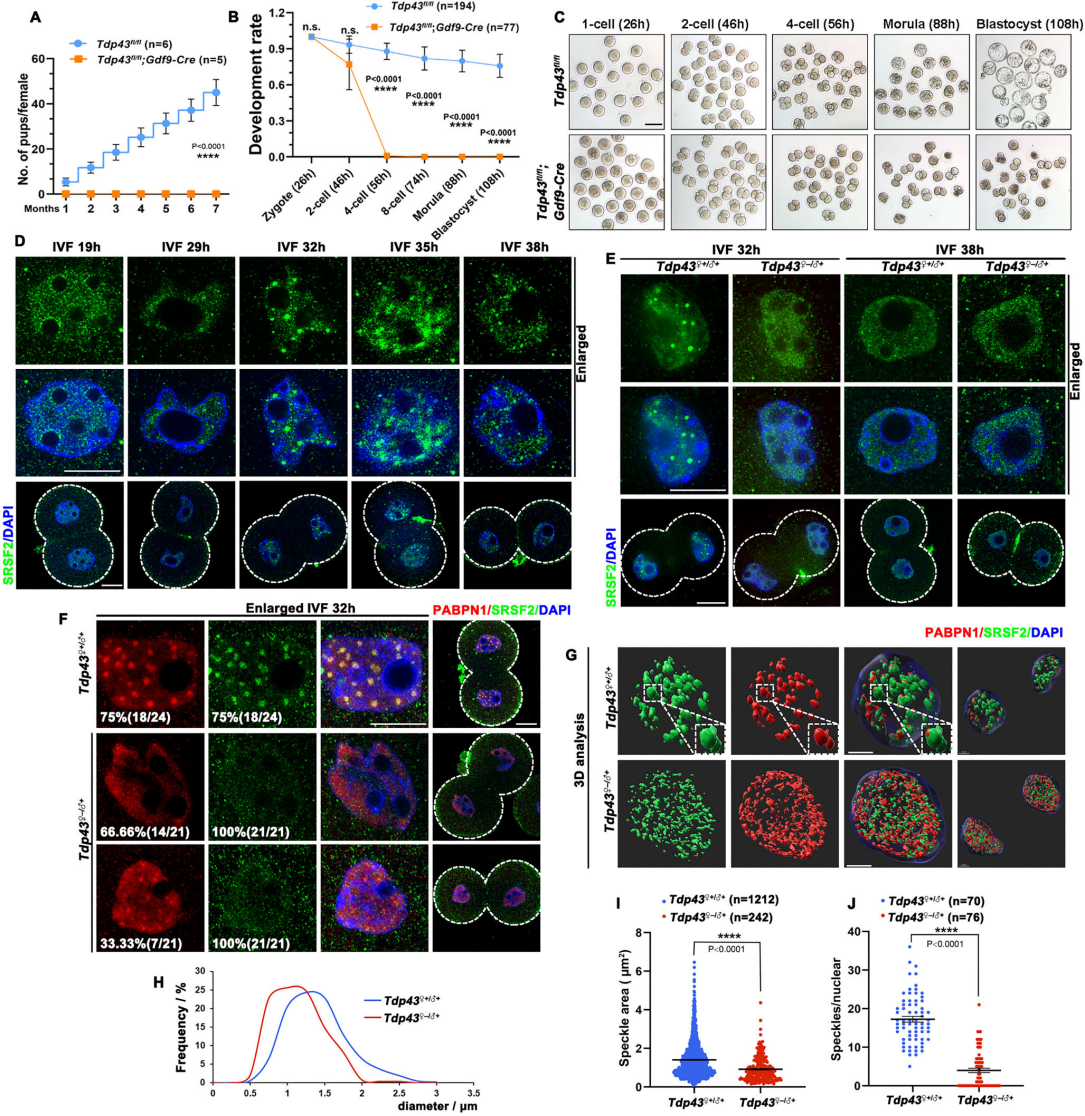
Deficiency of maternal TDP43 disrupts the assembly of NS in 2-cell embryos. (**A**) Cumulative numbers of pups per female, indicating the fertility of *Tdp43^fl/f^* and *Tdp43^fl/fl^; Gdf9-Cre* mice. *n* ≥ 4 females for each genotype. Data are presented as mean ± SEM. **** *P* < 0.0001 via two-tailed Student’s *t*-test. (**B**) The developmental rate of embryos was evaluated. Error bars, SEM. **** *P* < 0.0001 via two-tailed Student’s *t*-test. n.s. indicates non-significant. The number of embryos analyzed is indicated (*n*). (**C**) DIC images indicating the development of embryos obtained from 4-week-old *Tdp43^fl/fl^* and *Tdp43^fl/fl^; Gdf9-Cre* female mice mated with WT male mice. Zygotes, 2- and 4-cell embryos, morulae, and blastocysts were photographed at 26, 46, 56, 88, and 108 h after injection of hCG, respectively. (**D**) Immunofluorescence staining images indicating the progress of NS dynamic changes of 2-cell embryos at different time points after IVF. Scale bar = 20 μm. (**E**) Comparison of 2-cell NS assembly between control and knockout females at 32 h and 38 h post-fertilization. Scale bar = 20 μm. (**F**) Immunofluorescence images indicating the distribution of NS-associated proteins in 2-cell embryos at IVF 32 h of control and knockout females. Scale bar = 20 μm. (**G**) 3D model diagram of 2-cell embryos. Scale bar = 5 μm. (**H**) Quantitative statistics of changes in the size of NSs. (**I**) Quantification of the size of NSs. The number of analyzed speckles is indicated (*n*). Error bars, SEM. **** *P* < 0.0001 via two-tailed Student’s *t*-test. (**J**) Quantification of the number of NSs in 2-cell embryos. The number of nuclei analyzed is indicated (*n*). Error bars, SEM. **** *P* < 0.0001 via two-tailed Student’s *t*-test.

To study the *in vivo* function of TDP43, we generated *Tdp43* conditional knockout mice. Two LOXP sites were inserted, flanking exons 3 and 4 of *Tdp43* (*Tdp43^fl/fl^*) (Supplementary Fig. S2A). *Tdp43^fl/fl^* mice were crossed with *Gdf9-Cre* mice to knock out *Tdp43* in oocytes (*Tdp43^oo–/–^*) as early as the primordial follicle stage. The knockout efficiency of TDP43 was confirmed using western blotting and immunofluorescence (Supplementary Fig. S2B and C). A 7-month fertility test demonstrated that *Tdp43^fl/fl^;Gdf9-Cre* mice were sterile (Fig. 2A), indicating that TDP43 is essential for female fertility.

Moreover, germinal vesicle breakdown and polar body-1 (PB1) emission rates of TDP43-deficient oocytes were similar to those of control oocytes (Supplementary Fig. S2D and E). The TDP43-deficient oocytes were successfully fertilized (Supplementary Fig. S2F), indicating that the loss of TDP43 does not affect oocyte growth. Most of the *in vitro*-cultured embryos derived from *Tdp43^fl/fl^; Gdf9-Cre* mice (*Tdp43^♀–/♂+^*) successfully developed into 2-cell embryos, but few progressed beyond this stage (Fig. 2B and C). Collectively, these results revealed that maternal TDP43 is essential for mouse embryonic development, particularly at the 2- to 4-cell stages. This was consistent with the results from a previous report [34].

### Maternal TDP43 ensures the assembly of NSs in 2-cell stage embryos

Given the dynamic changes of NSs at the 2-cell stage, the control (subsequently labeled as *Tdp43^♀+/♂+^*) and *Tdp43^♀–/♂+^* embryos were collected at different time points post-fertilization to track the alternation of NS. Ovulated oocytes collected from the oviducts were IVF to synchronize the embryonic development processes. Immunofluorescence results indicated that NS proteins were uniformly distributed in the nucleus at IVF 19 h after the first cleavage was completed. Ten hours later, 2–3 pinhole-like condensates began to form (IVF 29 h). The number of NSs steadily increased and peaked at IVF 32 h. Finally, the condensates gradually merged and diffused at IVF 38 h (Fig. 2D). For further analyses of the NS assembly in 2-cell embryos, *Tdp43^♀+/♂+^* and *Tdp43^♀–/♂+^* embryos at IVF 32 h were applied. NS failed to assembly in 2-cell embryos of *Tdp43^♀–/♂+^*, while the NSs were present in the control (Fig. 2E). Additionally, NS proteins remained diffusive at later stages (Fig. 2E), confirming that the loss of TDP43 resulted in the failure of NS formation, rather than delayed formation.

Considering that the NS is a multi-protein complex, we co-stained for other important proteins in the NS, including PABPN1, ZC3H14, and SRRM2. All examined markers—whether PABPN1, ZC3H14, or SRRM2—were consistently co-localized with SRSF2 in *Tdp43^♀+/♂+^* embryos (Fig. 2F and G and Supplementary Fig. S3A and B), demonstrating an intact NS organization. However, the NS structures in over 70% of *Tdp43^♀–/♂+^* 2-cell embryos exhibited diffuse distribution when using SRSF2, PABPN1, ZC3H14, and SRRM2 as markers (Fig. 2F and G and Supplementary Fig. S3A and B). This widespread disruption across multiple core NS components collectively indicated that TDP43 regulates the assembly of NSs rather than one of the NS proteins. Both the size and number of NSs in *Tdp43^♀–/♂+^* 2-cell embryos were much smaller than those in the control (Fig. 2H–J). We divided the nucleus into four bins based on the speckle numbers in a single blastomere and observed a decrease in NS volume in all groups, regardless of the speckle density (Supplementary Fig. S3C).

Studies have demonstrated that SF3B1, an important splicing factor, becomes functional after phosphorylation, and the phosphorylated SF3B1 (pSF3B1) participates in the NS complex [15, 35]. Therefore, we determined the location and expression of SF3B1 and pSF3B1 and observed that TDP43 deletion had a minimal effect on the expression of SF3B1. However, its phosphorylation was blocked, and it could not assemble in the NS (Supplementary Fig. S3D–G). These results suggested that TDP43 is required for extensive remodeling of NSs after fertilization.

### TDP43 regulates NS directly rather than indirectly

**Figure 3.**
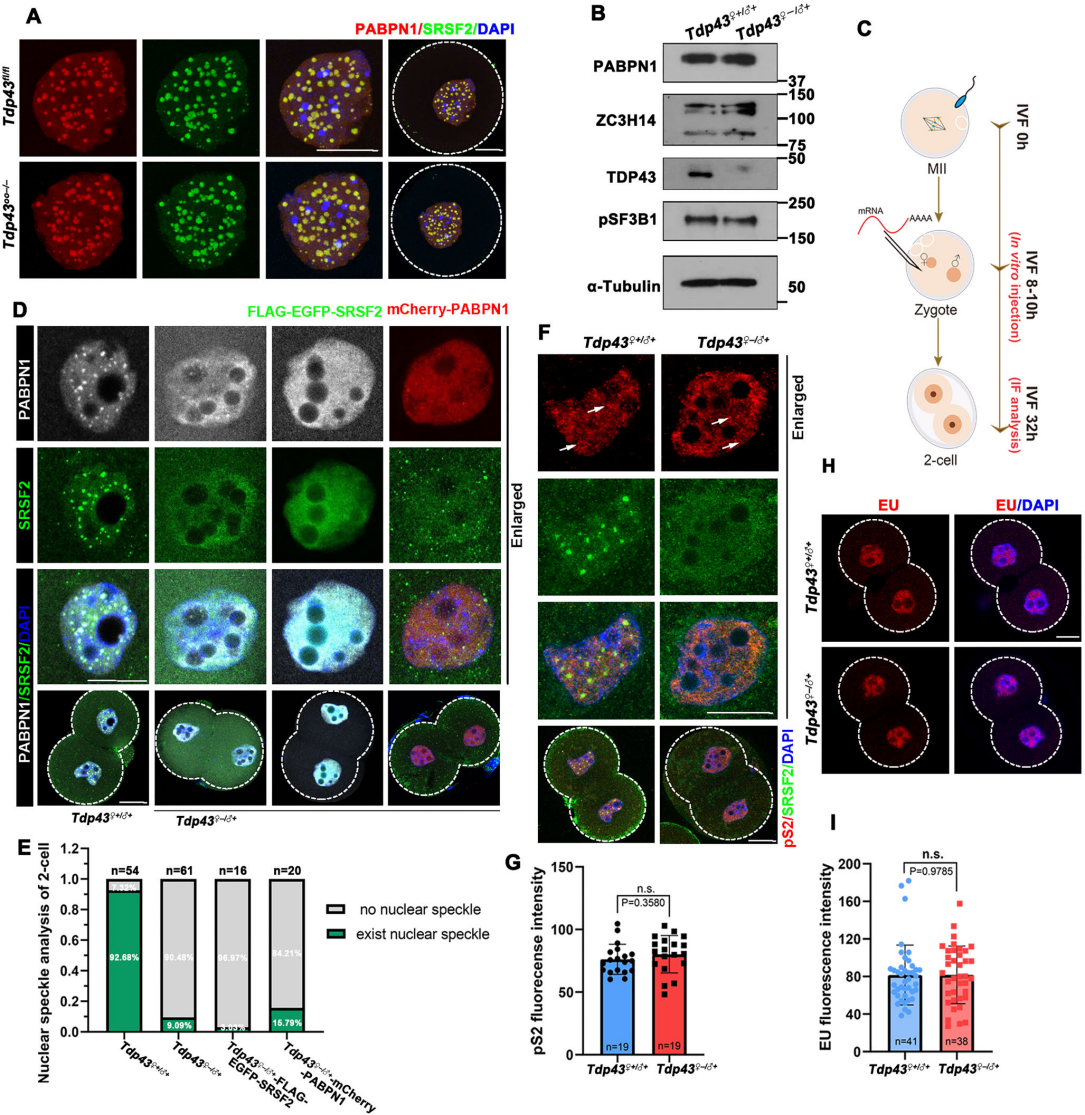
*Tdp43* deletion hardly affects the expression of NS proteins and transcription activity. (**A**) Representative images of 14-day growing oocytes indicating the NSs of the control and knockout females. Scale bar = 20 μm. (**B**) Western blot indicating the expression level of NS-associated proteins in control and knockout females. Total proteins from 200 2-cell embryos were loaded into each lane. (**C**) Schematic diagram providing the injection time and sample collection for immunofluorescence. (**D**) Immunofluorescence images indicating the effect of injection of *Flag-egfp-SRSF2* mRNA and *mCherry-Pabpn1* mRNA in *Tdp43^♀–/♂+^* zygotes on the assembly of NS in 2-cell embryos. The gray PABPN1 channels represent endogenous PABPN1 protein localization immunolabeled by antibodies. All green SRSF2 channels except those in column 3 represent endogenous SRSF2 localization via antibody detection. The green SRSF2 channel in column 3 displays exogenously expressed EGFP-SRSF2, while the red PABPN1 channel in column 4 indicates exogenously expressed mCherry-PABPN1. All 2-cell embryos were collected at IVF 32h. Scale bar = 20 μm. (**E**) Existence of NS in the indicated 2-cell embryos from panel (D). The number of analyzed 2-cell embryos is indicated (*n*). (**F**) Immunofluorescence images indicating the expression and assembly of transcription-related bodies based on phosphorylated RNA polymerase II CTD repeat YSPTSPS (pS2). White arrows indicate transcription-related condensates. Scale bar = 20 μm. (**G**) Quantification of fluorescent pS2 signals in panel (F). The number of analyzed 2-cell embryos is indicated (*n*). Error bars, SEM. n.s. means non-significant. (**H**) EU fluorescent staining indicating the transcriptional activity of 2-cell embryos from *Tdp43^♀+/♂+^* and *Tdp43^♀–/♂+^* females. (**I**) Quantification of EU fluorescent signals in panel (H). Error bars, SEM. n.s. means non-significant. The number of analyzed 2-cell embryos is indicated (*n*).

Although the interference of TDP43 with NS was significant in 2-cell embryos, it exerted little effect on oocytes (Fig. 3A). This indicates that TDP43 exhibits a stage-specific regulation of NS and that maternal TDP43 primarily functions during zygotic splicing activation. More importantly, western blot results demonstrated that the expression levels of PABPN1 and ZC3H14 in *Tdp43^♀–/♂+^* embryos were identical to those of the control (Fig. 3B). Furthermore, mRNAs encoding NS proteins (800 ng/µl) were injected into *Tdp43^♀–/♂+^* zygotes at IVF 6–8 h to confirm that NS assembly failure was a direct cause of *Tdp43* deletion. Embryos were collected and monitored for potential NS assembly in 2-cell embryos at IVF 32 h (Fig. 3C). When maternal TDP43 was absent, neither endogenous nor exogenously expressed NS proteins, i.e. FLAG-EGFP-SRSF2 or mCherry-PABPN1, formed speckles (Fig. 3D and E). These results indicated that the failure of NS assembly was not due to a deficiency in NS components, further confirming that TDP43 deletion interfered with the assembly of NSs rather than the expression of related proteins.

Multiple studies have demonstrated that NS assembly depends on transcription and that NSs fail to form when transcriptional activation is blocked [21]. Therefore, we sought to determine whether the effect of TDP43 on NSs relies on transcription. Phosphorylation of RNA polymerase II at serine-2 (pS2) indicated that transcription-related bodies were discretely located and did not co-localize with NSs in 2-cell embryos (Fig. 3F). Furthermore, the transcription activity detected through the results of the EU incorporation assay and pS2 fluorescence quantification indicated that TDP43 deletion did not affect transcription in embryos (Fig. 3G–I). These results further supported the hypothesis that TDP43 regulates NS assembly directly rather than indirectly through transcription.

### The nuclear localization and RNA-binding ability of TDP43 are required for NS assembly

**Figure 4.**
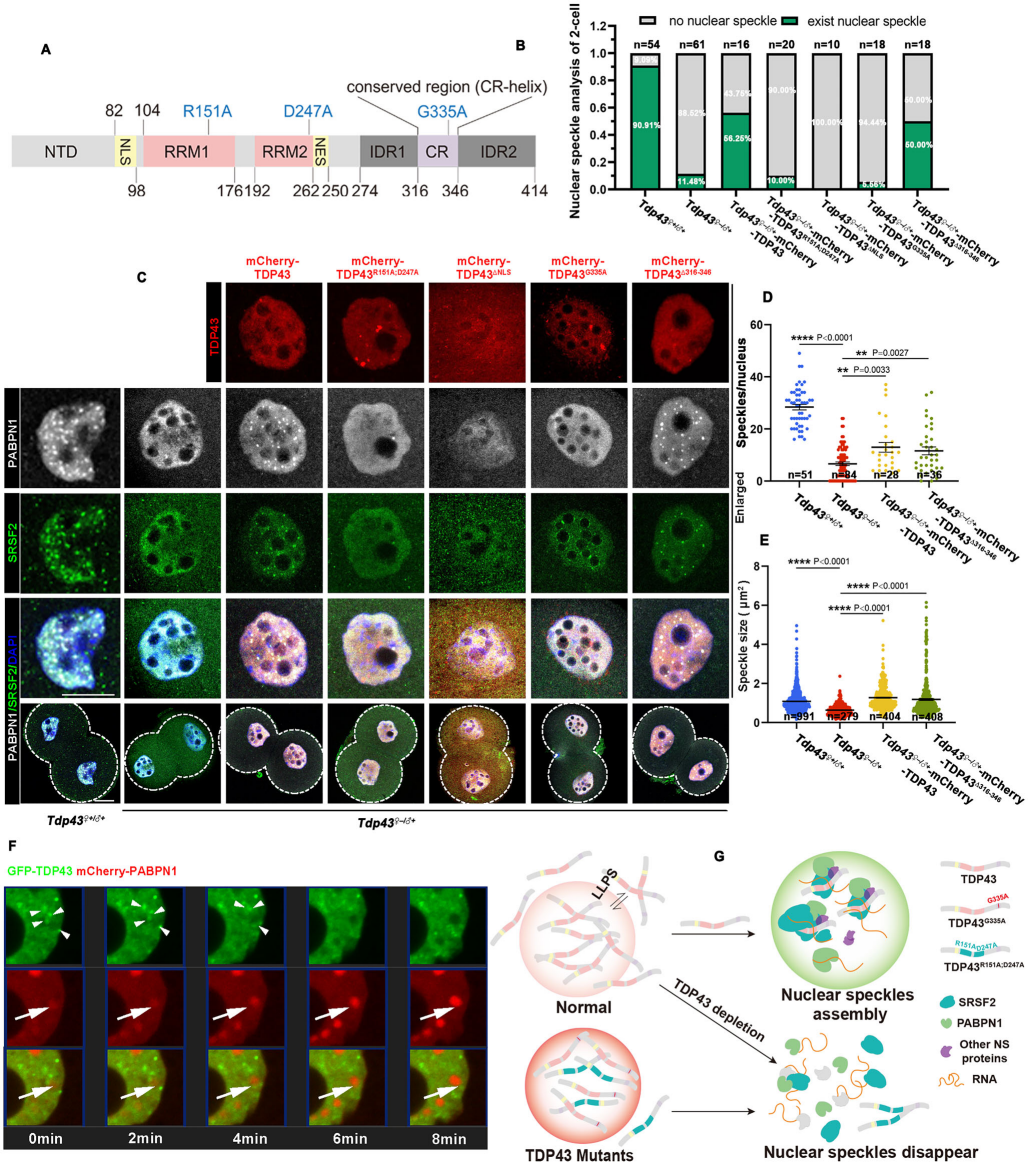
Supplementing TDP43 in *Tdp43^♀–/♂+^* zygotes restored the assembly of NS in 2-cell embryos. (**A**) Diagram providing the structure of TDP-43, including an N-terminal domain, a nuclear localization signal (NLS), a nuclear export signal, two RNA recognition motifs (RRM1 and RRM2), two intrinsically disordered regions (IDR1, IDR2), and an intervening conserved region (CR helix). (**B**) Quantification of NS existence in 2-cell embryos from panel (C). The number of analyzed 2-cell embryos is indicated (*n*). (**C**) Immunofluorescence images indicating the recovery of NSs in 2-cell after supplementation with TDP43 and its mutant in knockout zygotes. All 2-cell embryos were collected at IVF 32h. Scale bar = 20 μm. (**D**) Quantification of the number of NSs in 2-cell embryos. The number of nuclei analyzed is indicated (*n*). Error bars, SEM. **P < 0.01, ****P < 0.0001 via two-tailed Student’s *t*-test. (**E**) Quantitative statistics of changes in the size of NSs. Error bars, SEM. ****P < 0.0001 via two-tailed Student’s *t*-test. The number of analyzed speckles is indicated (*n*). (**F**) Time-lapse images of the dynamic changes of GFP-TDP43 and mCherry-PABPN1 in the nucleus of growing oocytes. Droplet-like TDP43 is indicated using a white triangle. (**G**) A diagram indicating a condensation-deficient state TDP43 rather than a condensation-preserving state TDP43 with less dynamics can help the assembly of NS.

We constructed mCherry-tagged TDP43 expression plasmids for the subsequent experiments. As a nuclear-localized protein, TDP43 primarily functions in the nucleus. Therefore, we constructed *Tdp43^ΔNLS^* as a negative control. Destroying a single salt bridge between Arg151 and Asp247 in TDP43 caused more than a 37-fold decrease in RNA affinity [36]. Therefore, we constructed *Tdp43^R151A;D247A^* to evaluate the importance of the RNA-binding ability of TDP43 in NS formation (Fig. 4A). In 293T cells, mCherry-TDP43^ΔNLS^ was localized in the cytoplasm, while mCherry-TDP43^R151A;D247A^ was nuclear-localized (Supplementary Fig. S4A). Notably, mCherry-TDP43^R151A; D247A^ formed condensates in 293T cells (Supplementary Fig. S4A). These TDP43 mutants did not affect NS formation or cause abnormal NS morphology in 293T cells (Supplementary Fig. S4A).

Different from supplementing SRSF2 or PABPN1, NSs were reformed in *Tdp43^♀–/♂+^* 2-cell embryos after *Tdp43* mRNA microinjection (Fig. 4B and C). Although the sizes of restored NSs were identical to those in *Tdp43^♀+/♂+^* 2-cell embryos, fewer speckles were present in *Tdp43^♀–/♂+^* embryos (Fig. 4D and E). This indicated that the rescue effect was limited and did not fully complement the damage caused by the loss of maternal TDP43. As expected, supplementing TDP43^ΔNLS^ was ineffective, as it cannot enter the nucleus to function (Fig. 4B and C). Although the expression of TDP43^ΔNLS^ was verified before, a small amount of TDP43^ΔNLS^ was still present in the nucleus in the *Tdp43^♀–/♂+^* 2-cell embryos (Fig. 4C), likely due to the effect of other TDP43-interacting proteins residing in the nucleus. Moreover, expressing TDP43^R151A;D247A^ failed to induce NS assembly in *Tdp43^♀–/♂+^* embryos (Fig. 4B and C). These results revealed that TDP43 directly affects NS assembly, which is dependent on its nuclear localization and RNA-binding ability.

### Condensation-deficient TDP43 rather than condensation-preserving TDP43 assists NS assembly in early embryos

TDP43 undergoes phase separation depending on the weak homomeric contacts between two disordered regions (IDR1 and IDR2) and a short conserved region (CR) [23]. Mutations in the C-terminal low-complexity domain strengthen or weaken TDP43 aggregation [37–39]. Therefore, we investigated the influence of TDP43 phase separation on NS assembly.

TDP43^G335A^ promotes condensation by extending the CR helix, while CR-deletion (TDP43^Δ316–346^) disrupts TDP43 phase separation [33, 40]. We constructed these two mutants and verified their localization and expression in 293T cells and oocytes (Fig. 4A and Supplementary Fig. S4A and B). Both expressed well in the nucleus, and TDP43^Δ316–346^ completely diffused in the nucleoplasm, while TDP43^G335A^ formed discrete speckles in somatic cells and 2-cell embryos (Fig. 4C and Supplementary Fig. S4A). TDP43^G335A^ with enhanced phase separation did not rescue the formation of NSs when microinjected into *Tdp43^♀–/♂+^* zygotes. In contrast, TDP43^Δ316–346^ that could not undergo phase separation induced NS recovery in the same manner as that by TDP43 (Fig. 4B and C). Furthermore, live cell imaging revealed that droplet-like TDP43 was enriched near the NS in advance. Additionally, NS gradually formed and became larger as the droplet-like TDP43 gradually converted into a diffuse state (Fig. 4F). These results indicated that the condensation-deficient state, rather than the condensation-preserving state, of TDP43 was the active form that exerts its function. Based on the dynamic changes in TDP43 and NSs observed in 2-cell embryos, combined with our results from the rescue experiments, we propose that TDP43 and its phase separation capacity are essential for NS regulation. TDP43 can undergo dynamic alterations through LLPS. Specifically, some condensation-deficient TDP43 is released during ZSA, which facilitates NS assembly. Both maternal TDP43 deficiency and overexpression of condensation-preserving TDP43 mutants (TDP43^G335A^ and TDP43^R151A;D247A^) lead to NS assembly failure in 2-cell embryos, as demonstrated (Fig. 4G).

### Excessive TDP43 is harmful to NS assembly and embryo development

As TDP43 and TDP43^Δ316–346^ could mediate NS re-formation in *Tdp43^♀–/♂+^* 2-cell embryos, we cultured these injected embryos to observe the *in vitro* development. As expected, embryos that could not reform NSs after complementing TDP43^ΔNLS^ or TDP43^R151A;D247A^ still arrested at the 2-cell stage like the *Tdp43^♀–/♂+^* embryos (Fig. Supplementary S5A and B). However, neither TDP43 nor TDP43^Δ316–346^ complementation could rescue embryonic development (Supplementary Fig. S5A and B). Therefore, we tested whether excessive TDP43 is harmful to embryonic development by microinjecting mRNAs encoding wild-type (WT) or mutated TDP43 into WT zygotes.

**Figure 5.**
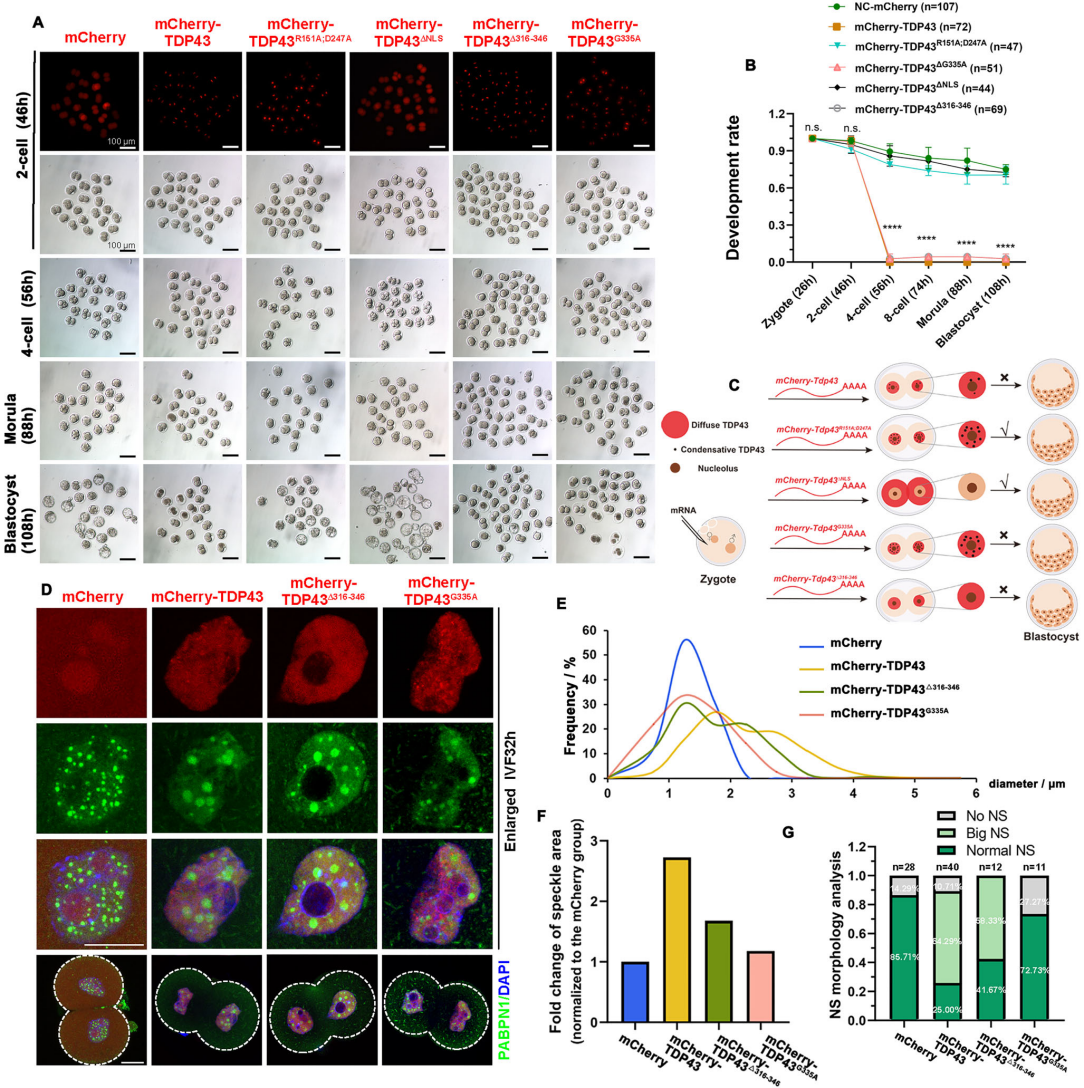
Excessive TDP43 causes abnormal enlargement of NSs and embryonic arrest. (**A**) Representative images of embryos injected with *Tdp43* and its mutant mRNA at different development stages. Fluorescence images indicating the expression efficiency of *in vitro* transcribed mRNA. All embryos were microinjected at 6–8 h after fertilization. Scale bar = 100 μm. (**B**) The developmental rate of embryos was evaluated from panel (A). Data are presented as mean ± SEM. ****P < 0.0001 via two-tailed Student’s *t*-test. n.s. indicates non-significant. The number of embryos analyzed is indicated (*n*). (**C**) Schematic diagram indicating the localization of TDP43 and its mutants in 2-cell embryos after injection of corresponding mRNA into zygotes as well as their impact on early embryonic development. (**D**) Immunofluorescence images indicating the status of NSs in 2-cell embryos after injection of TDP43 and its mutants in the zygote. Scale bar = 20 μm. (**E, F**) Statistical analysis of the size of NSs in 2-cell embryos from panel (D). The number of analyzed NSs is indicated (*n*). (**G**) Statistics of NSs existence in 2-cell embryos from panel (D). The number of analyzed 2-cell embryos is indicated (*n*).

Western blot analysis indicated that the expression level of exogenous TDP43 was much higher than that of endogenous TDP43 (Supplementary Fig. S6A and B). Although all embryos developed into 2-cells, the embryos microinjected with *Tdp43* mRNAs could not develop into 4-cells, while the embryos microinjected with *Tdp43^R151A;D247A^* or *Tdp43^ΔNLS^* mRNAs successfully developed into blastocysts similar to those of the control group (Fig. 5A and B). This indicated that excessive TDP43 is harmful to embryonic development, depending on its nuclear localization and RNA-binding ability. Moreover, ectopic expression of *Tdp43^G335A^* or *Tdp43^Δ316–346^* also caused 2-cell arrest (Fig. 5A and B). These results are summarized in Fig. 5C. TDP43 and TDP43^Δ316–346^, but not TDP43^G335A^, significantly promoted excessive expansion of NSs, indicating aberrant NS assembly (Fig. 5D). The diameters of the NSs in the control were ~1.3 μm, and a large proportion of the NS diameters of TDP43^G335A^ were also within this range (Fig. 5E). However, two peaks appeared in the diameters of TDP43 and TDP43^Δ316–346^. The first wave overlapped with the control, but the second wave revealed that the diameter of their NSs increased to 2–3 μm (Fig. 5E), indicating they caused some but not all NSs to be larger. Consistently, we observed that excessive TDP43 and TDP43^Δ316–346^ increased the average NS size by 2.7- and 1.7-fold, respectively (Fig. 5F and Supplementary Fig. S7A). According to the peak distribution of diameter, we define NSs with a diameter larger than 2.3 μm as abnormally large. TDP43 or TDP43^Δ316–346^ overexpression caused the formation of abnormally large NSs in more than half of the embryos (Fig. 5G); this may be responsible for embryo arrest. After subdividing the cell nuclei according to the number of speckles, we determined that TDP43 and TDP43^Δ316–346^ caused larger NSs in almost all types of nuclei. In contrast, TDP43^G335A^ slightly affected NS size, and some NSs of TDP43^G335A^ even became smaller (Supplementary Fig. S7B and C). Excessive TDP43 did not affect the number of NSs, while TDP43^G335A^ led to fewer NSs, and TDP43^Δ316–346^ led to more NSs (Supplementary Fig. S7D). To further ensure that the observed NS abnormalities were not coincidental, we performed comprehensive detection of multiple NS markers following microinjection of TDP43 and its mutant variants. Our results reveal that overexpression of both TDP43 and TDP43^Δ316–346^ consistently induces abnormally enlarged morphology in NSs, as evidenced by the markers PABPN1, SRRM2, and SRSF2 (Fig. 5D and Supplementary Fig. S7E and F). These pathological alterations in NS morphology and abundance revealed that the LLPS state of TDP43 is an important factor in NS regulation. Moreover, when injected TDP43 was adjusted to the same level as endogenous TDP43 expression, there was almost no effect on embryonic development (Supplementary Fig. S6C–E). These results indicated that dose-dependent regulation of TDP43 plays a key role in embryonic development.

Additionally, we observed that exogenous PABPN1 condensates became abnormally large (62.50%) when TDP43 and NS protein PABPN1 were co-overexpressed (Supplementary Fig. S8A). However, the size of the NSs in the 2-cell embryos remained normal when PABPN1 or mCherry was overexpressed alone (Supplementary Fig. S8B and C). In addition, the PABPN1-overexpressing and control embryos developed normally (Supplementary Fig. S8D and E). These results indicated that TDP43 is a specific regulatory factor in NS assembly during ZSA.

### Both TDP43 deletion and overexpression lead to transcriptome dysregulation in 2-cell embryos

**Figure 6.**
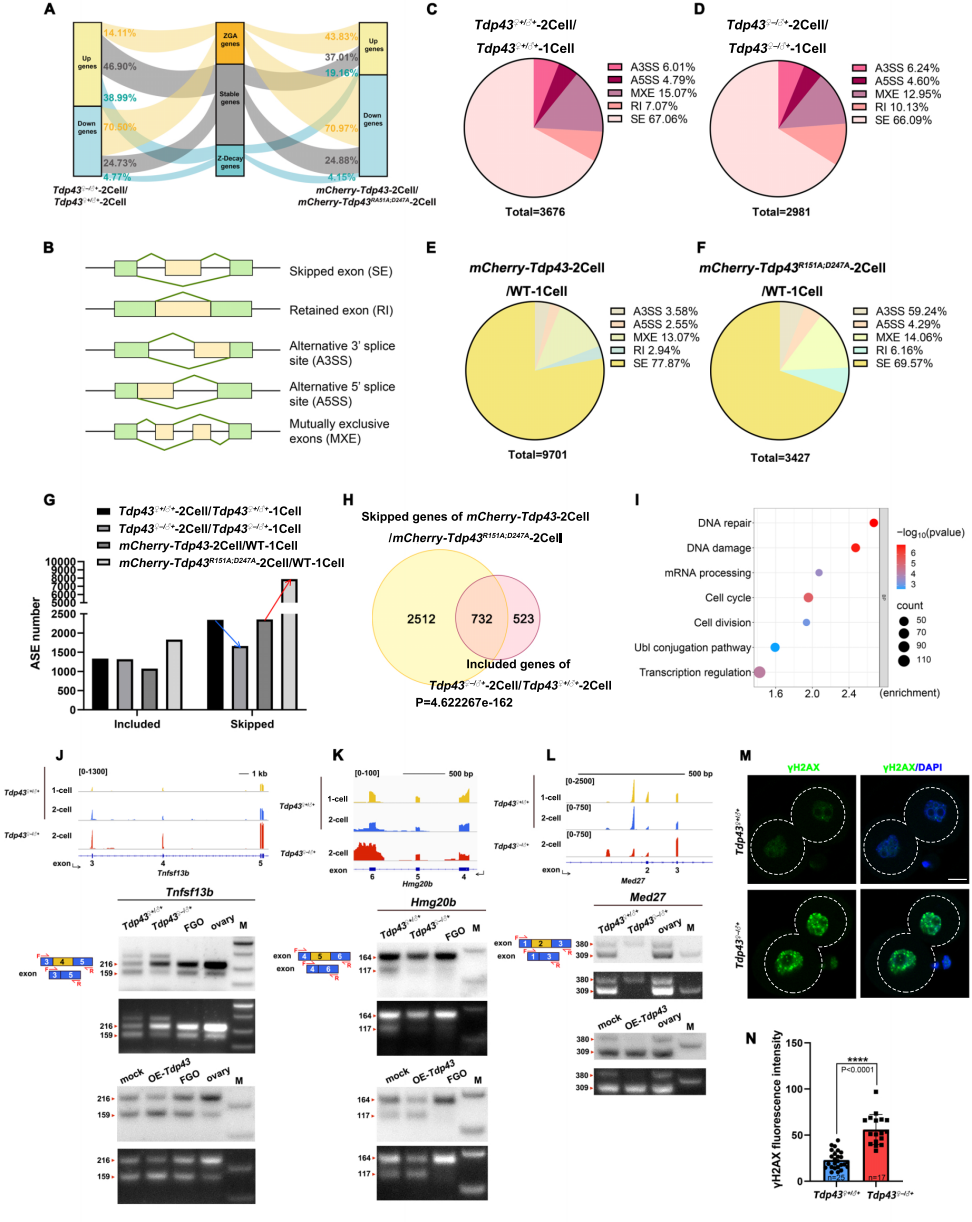
ASE analysis of TDP43-deficient and TDP43-overexpressing embryos during ZGA. (**A**) Sankey diagram indicating the global changes of transcripts during zygote to 2-cell among normal, TDP43-deficient, and TDP43-overexpressing embryos. (**B**) Diagram indicating five major types of ASEs. Constitutively expressed exons and alternatively spliced exons are indicated in green and yellow, respectively. (C–F) Pie charts indicating the number and ratio of different ASEs in normal (**C**), TDP43-deficient (**D**), TDP43-overexpressing (**E**), and TDP43^R151A;D247A^-overexpressing (**F**) embryos from zygote to 2-cell stage. (**G**) The ASE number of skipped events and included events from different groups during ZGA. (**H**) Venn diagram indicating shared genes that skipped ASEs upon *Tdp43* overexpression and included events in the *Tdp43*-deficient group. (**I**) Gene Ontology analysis indicates the potential functions of the 732 genes in panel (H). (**J**–**L**) The IGV diagrams indicate abnormal exon skipping events in 2-cell embryos during ZGA after *Tdp43* deletion. The schematic diagram in the lower left corner indicates two isoforms of transcripts after corresponding exon skipping during the ZGA process. F refers to the forward primer, and R refers to the reverse primer. Validation of exon skipping using PCR is indicated below. The cDNAs were prepared from *Tdp43^♀+/♂+^, Tdp43^♀-/♂+^*, and *Tdp43-*overexpressed (labeled as “OE-*Tdp43*”) 2-cell embryos as indicated. WT ovary and FGO cDNAs were applied as controls. “M” indicates marker. (**M**) Representative images indicating the expression signal of γH2AX. Scale bar = 20 μm. (**N**) Quantification of γH2AX fluorescent signals in panel (M). The number of analyzed 2-cell embryos is indicated (*n*). Error bars, SEM. ****P < 0.0001 via two-tailed Student’s *t*-test. All analysis of ASEs in Fig. 7 was defined as FDR < 0.05 and |ILD| ≥ 0.1. FDR, false discovery rate; ILD, inclusion level difference.

We collected *Tdp43*-deleted or -overexpressed embryos for transcriptome sequencing (Smart-seq2) analyses. Heatmaps indicated that all replicates were highly correlated, confirming the reliability of the data (Supplementary Fig. S9A and B). The volcano plot demonstrated that *Tdp43* deletion barely affected the global transcript levels in zygotes (Supplementary Fig. S9C). In contrast, many differentially expressed genes (DEGs) were detected at the 2-cell stage in both *Tdp43*-deletion and -overexpressed embryos (Supplementary Fig. S9D and E). *Tdp43*-deletion resulted in the downregulation of 2095 transcripts, of which 70.50% (1477/2095) belonged to ZGA genes (Supplementary Fig. S9F). It also resulted in upregulation of 2511 transcripts, of which 37.51% (942/2511) belonged to maternal transcripts that were removed after ZGA (Z-decay transcripts) (adjusted *P-*value < 0.05; |log_2_ Fold change| ≥1) (Supplementary Fig. S9G). qRT-PCR confirmed that *Tdp43*-deletion led to severe transcriptional dysregulation during ZGA (Supplementary Fig. S9H). Furthermore, TDP43 overexpression downregulated 970 transcripts, of which 41.44% (402/970) overlapped with genes downregulated by maternal *Tdp43* knockout (Supplementary Fig. S9I), indicating the importance of TDP43 in regulating ZGA gene expression. Therefore, either deletion or overexpression of TDP43 led to gene expression abnormalities at the 2-cell stage, including failure of ZGA gene expression, failure of maternal mRNA degradation, and abnormal upregulation or downregulation of transcripts that were stably maintained during the MZT (Fig. 6A).

### TDP43 is required for zygotic splicing activation in 2-cell embryos

Considering that TDP43 abnormalities lead to the dysregulation of NS assembly, we investigated how this dysregulation affects ZSA. ASEs generally consist of five types that include skipped exon (SE), retained intron (RI), alternative 3′- and 5′-splice sites (A3SS and A5SS), and MXE (Fig. 6B). We used rMATS to analyze ASEs as previously described [41, 42]. ASEs were defined as FDR < 0.05, and |IncLevelDifference| ≥ 0.1. There were 3676 ASEs during the normal development from the zygotes to 2-cell embryos. Over half of the events were SEs, while the number of ASEs in *Tdp43^♀–/♂+^* embryos dropped to 2981 during this period (Fig. 6C and D). The opposite trend was observed in embryos overexpressing TDP43: 9701 ASEs during zygote to 2-cell development (Fig. 6E). Overexpression of RRM-mutated TDP43 did not significantly affect ASEs (Fig. 6F and Supplementary Fig. S10A), which supported our hypothesis that TDP43 relies on RRMs for its functions.

Splicing events between two groups can be categorized into two types: inclusion and skipping events. Volcano plots indicated that skipping events accounted for 63.74% (2343/3676) during normal ZGA (Supplementary Fig. S10B). During the zygote-to-2-cell transition, the *Tdp43*-deletion group contained ~1300 inclusion events (similar to the control group); however, there were 681 fewer skipping events than in the control group (Fig. 6G and Supplementary Fig. S10B and C). In contrast, the TDP43-overexpressing group exhibited a 5529 increase in skipping event counts compared to the control group (Fig. 6G and Supplementary Fig. S10D). These results indicated that TDP43 regulates pre-mRNA processing during ZGA, mainly by facilitating exon skipping. Furthermore, the skipping progress between the knockout/control group and the TDP43-overexpressing/TDP43^R151A;D247A^-overexpressing group exhibited the opposite trend, regardless of the ASE type (Supplementary Fig. S10E). Collectively, these results confirmed that the expression level of TDP43 is crucial for determining the balance of ZSA, which primarily refers to the skipping process and exhibits no clear preference for splicing types.

Transcripts with excessive skipping in the TDP43 overexpression group and transcripts with abnormal inclusions in the TDP43 deletion group were compared to investigate whether TDP43 deletion and overexpression modulate the splicing of the same group of transcripts. In total, 732 transcripts were found to be jointly regulated (Fig. 6H). Gene ontology analysis showed that transcripts with abnormal splicing were responsible for important pathways related to development, such as transcriptional regulation, cell cycle, DNA damage, and cell division (Fig. 6I). Integrative genomics viewer (IGV) snapshots showed splicing changes in representative genes, including *Tnfsf13b, Hmg20b*, and *Med27* (Fig. 6J–L). We designed corresponding primers for PCR analysis to verify the results of AS analysis. These transcripts underwent abnormal splicing under TDP43 dysregulation conditions, and the opposite trend was detected in *Tdp43* knockout and overexpressing cells (Fig. 6J–L). Additionally, a group of DNA damage and repair gene-coding transcripts were aberrantly spliced upon TDP43 deficiency and excess (Fig. 6I). Accumulation of γH2AX, a DNA damage indicator, was detected in *Tdp43^♀–/♂+^* 2-cell embryos (Fig. 6M and N), which may be another cause of embryo arrest.

### TDP43-dependent ZSA is essential for the maintenance of transcriptome homeostasis and totipotency-pluripotency conversion

**Figure 7.**
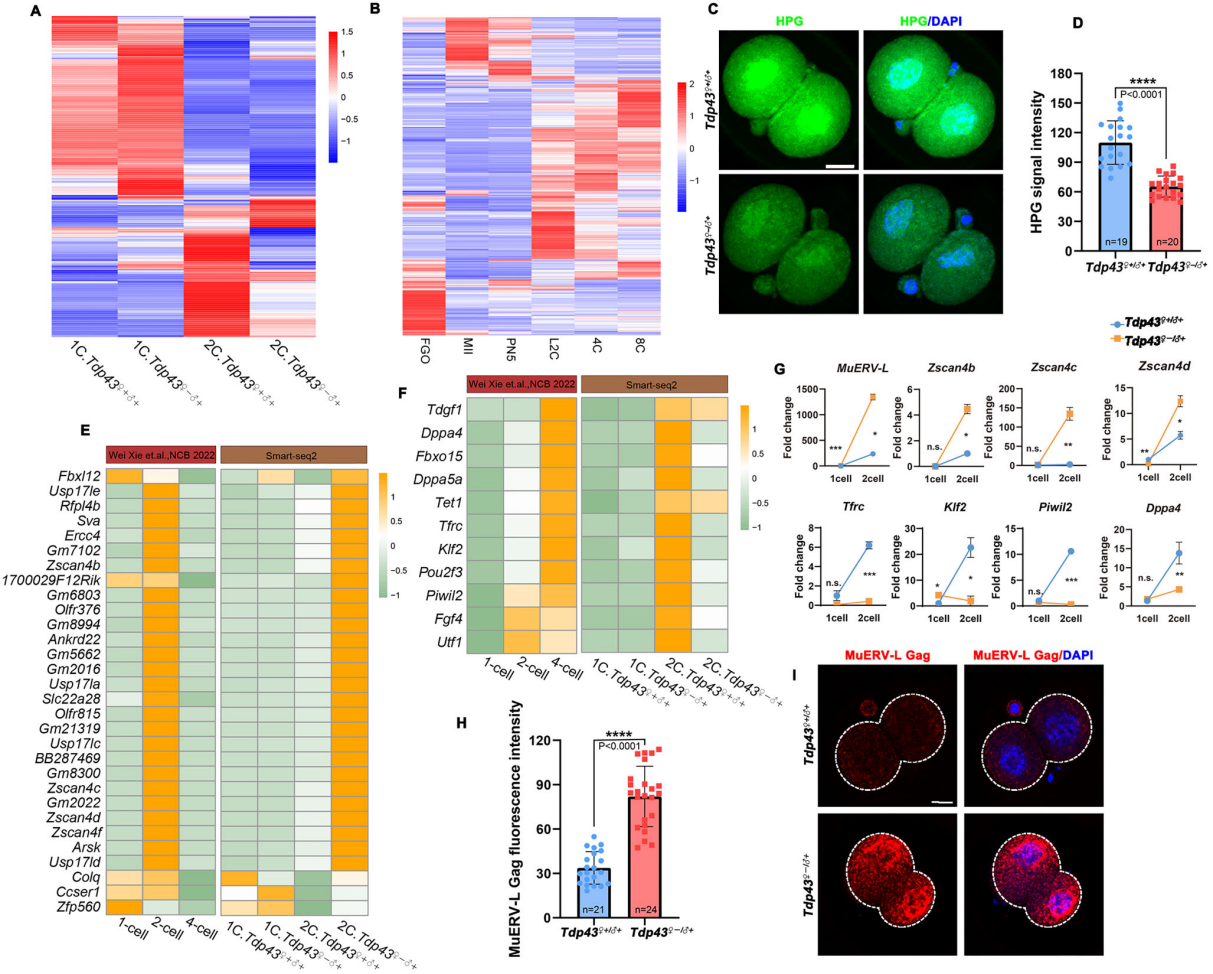
TDP43 deficiency leads to transcript dysregulation and disrupts the expression of totipotency and pluripotency genes in 2-cell embryos. (**A**) Heatmap providing the relative expression levels of genes with different ASEs between control and TDP43-deficient 2-cell embryos. (**B**) Heatmap indicating the relative translation levels of genes with different ASEs between control and TDP43-deficient 2-cell embryos. (**C**) HPG staining results indicating protein translation status of 2-cell embryos. Scale bar = 20 μm. (**D**) Quantification of HPG immunofluorescence intensity in panel (**C**). The number of analyzed 2-cell embryos is indicated (*n*). Error bars, SEM. ****P < 0.0001 via two-tailed Student’s *t*-test. (E, F) Heatmap of the relative expression levels of representative totipotent (**E**) and pluripotent genes (**F**) in 2-cell embryos. The lists of totipotent and pluripotent genes were derived from[44]. (**G**) RT-qPCR validation of the expression levels of totipotent and pluripotent genes in 2-cell embryos. Error bars, SEM. *P < 0.05, **P < 0.01, ***P < 0.001 via two-tailed Student’s *t*-test. n.s. indicates non-significant. (**H**) Quantification of MuERV-L Gag signal in panel (I). The number of analyzed 2-cell embryos is indicated (*n*). Error bars, SEM. ****P < 0.0001 via two-tailed Student’s *t*-test. (**I**) Representative images indicating the expression signal of MuERV-L Gag. Scale bar = 20 μm.

Subsequently, we analyzed the fate of these abnormally spliced transcripts. Most abnormal transcripts exhibited little difference in expression levels between *Tdp43*-deletion and control groups, with 21.62% downregulated (Fig. 7A). We speculated that this was the result of splicing errors leading to the activation of mRNA degradation pathways such as nonsense-mediated mRNA decay. Additionally, ribo-lite data analysis [43] indicated that most abnormally spliced transcripts were translationally activated during the embryonic development, with high translation levels in the 2-cell to 8-cell stages (Fig. 7B). However, the HPG assay showed that the translation level in *Tdp43^♀–/♂+^* 2-cell embryos was significantly reduced (Fig. 7C and D), indicating that these abnormally spliced transcripts could not be translated into proteins.

Splicing is essential for *in vitro* stem cell fate determination and early embryonic development. Treatment of embryonic stem cells (ESCs) with the splicing inhibitor pladienolide B drives the pluripotent-to-totipotent transition [44]. Considering that TDP43 deficiency leads to NS defects and ZSA failure, the correlation among TDP43, NS, splicing, and cell fate conversion needs to be investigated. Most totipotent genes, such as the *Zscan4s* family, the *Usp17ls* family, *Ercc4*, and *Sva*, typically increased in expression from the zygote to the 2-cell stage and decreased after the 4-cell stage [43] (Fig. 7E). The expression of classic pluripotency genes, such as *Dppa4, Dppa5a, Foxo15, Tet1*, and *Klf2*, gradually increased during early embryonic development (Fig. 7F). However, the expression of totipotency genes in *Tdp43^♀–/♂+^* 2-cell embryos was abnormally increased, while the pluripotency genes were suppressed (Fig. 7E and F). RT-qPCR results confirmed the upregulation of totipotency-related transcripts and downregulation of pluripotency-related transcripts in *Tdp43^♀–/♂+^* embryos (Fig. 7G). Immunofluorescence results consistently showed that the expression level of totipotency marker MuERV-L was elevated in *Tdp43^♀–/♂+ ^*2-cell embryos (Fig. 7H and I). These results suggested that ZSA, mediated by maternal TDP43, plays an important role in the conversion of totipotency to pluripotency in embryos.

### TDP43 can bind to both NS-associated proteins and RNAs

**Figure 8.**
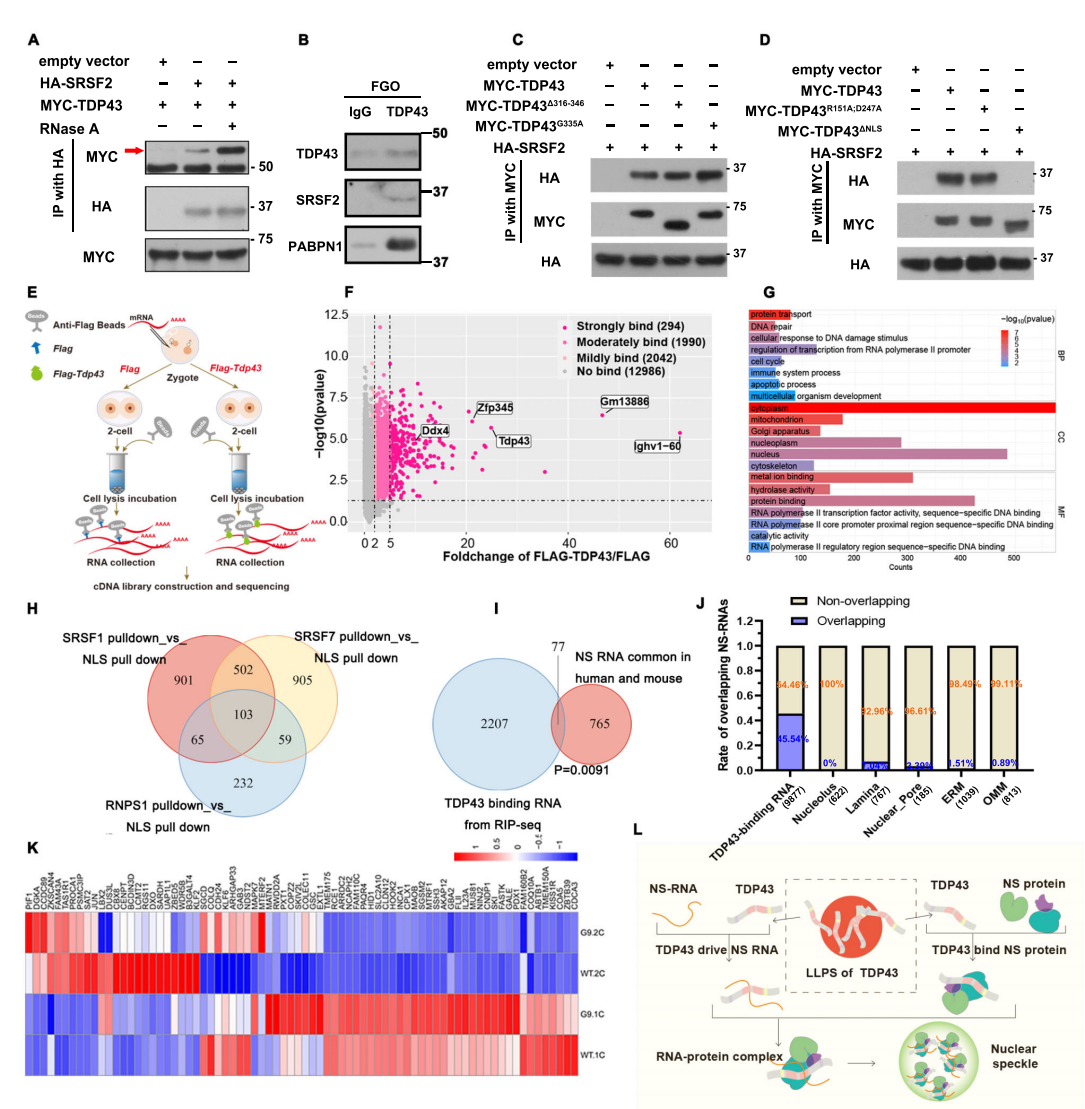
TDP43 could bind NS-associated proteins and RNAs. (**A**) Co-IP assay revealed that TDP43 binds directly to NS proteins. (**B**) Endogenous immunoprecipitation results of TDP43 in GV oocytes. Western blot indicating the binding of IP products to specific antibodies. Rabbit IgG isotype was applied as the negative control. (**C, D**) Co-IP assay revealed the interaction of TDP43 and its mutants with the NS protein, SRSF2. IP was performed using anti-MYC beads. (**E**) Schematic diagram providing the process of TDP43 RIP-seq library construction. WT zygotes injected with *Flag* and *Flag-Tdp43* mRNAs, respectively, were cultured to develop into the late 2-cell stage. Samples were lysed, and the RNAs were enriched by anti-FLAG beads and subjected to library construction and sequencing. (**F**) Volcano plots indicating different binding levels of TDP43 to transcripts in 2-cell embryos based on RIP-seq data. Under the premise of *P*-value < 0.05, the transcripts with FLAG-TDP43/FLAG ≥ 5 were designated as strongly bound to TDP43, the transcripts with ≥ 2 FLAG-TDP43/ FLAG < 5 as moderately bound to TDP43, the transcripts with ≥ 1.5 FLAG-TDP43/ FLAG < 2 as mildly bound to TDP43, and the others as not bound to TDP43. (**G**) Gene Ontology analysis indicated the potential functions of the strongly and moderately TDP43-binding transcripts. (**H**) Venn diagram indicating 2 767 human NS-associated transcripts pulled down by three important NS proteins that included SRSF1, SRSF7, and RNPS1. (**I**) Venn diagram depicting the overlap between TDP43-bound RNAs in mouse embryos (this study) and NS RNAs that are shared in humans and mice. (**J**) Correlation analysis between NS-associated RNAs and RNAs associated with other subcellular structures. In 293T cells, RNAs with an average binding value > 2.5 in iCLIP-seq data were defined as TDP43-binding targets. RNAs associated with other subcellular structures were identified based on a log₂ fold change ≥ 1. The values below indicate the total RNA counts in each group. (**K**) Heatmap indicating the relative expression levels of the shared transcripts in panel (I). (**L**) Schematic diagram showing the potential process of TDP43 regulating NS assembly.

NS is an RNA–protein complex with highly intricate composition that poses significant challenges for study. To explore the role of TDP43 in the NS assembly process, we used immunoprecipitation (IP) to verify the binding of TDP43 to the NS protein. The results showed that TDP43 could bind to SRSF2, and when treated with RNase A, the binding between TDP43 and SRSF2 was not weakened (Fig. 8A), suggesting that this binding was a direct action independent of RNA. Endogenous immunoprecipitation (endo-IP) of FGOs consistently revealed that TDP43 interacted with SRSF2 and PABPN1 (Fig. 8B), indicating that the binding of TDP43 to the NS protein is conserved. Both TDP43^G335A^ and TDP43^Δ316–346^ could bind to SRSF2 as TDP43, indicating CR is not the key domain for TDP43 affinity towards NSs (Fig. 8C). Moreover, TDP43^R151A;D247A^ did not affect its binding to NS, indicating that RRM is not the key site for the binding of TDP43 to NS (Fig. 8D). Notably, TDP43^ΔNLS^ could not bind to NS protein (Fig. 8D), supporting our hypothesis that the NS regulatory function of TDP43 can only take place in the nucleus. These results indicated that TDP43 directly binds to the NS protein, suggesting a potential role for TDP43 in NS assembly.

Studies have reported that RNA is necessary for NS formation [21], and we observed that supplementing with TDP43^R151A;D247A^ failed to induce NS assembly in *Tdp43^♀–/♂+^* embryos, leading us to question whether TDP43 helps NS assembly through RNAs. Therefore, RNA-immunoprecipitation sequencing (RIP-seq) was performed using mouse 2-cell embryos as presented schematically (Fig. 8E). The putative TDP43-binding RNAs were further classified into three categories (strong, moderate, and mild) according to the fold change of in FLAG-TDP43/FLAG (*P*-value < 0.05) (Fig. 8F). We believe that the strongly and moderately bound RNAs were credible. There were 2284 in total, including *Tdp43* and its pseudogene *Gm13886*, which is consistent with a previous report showing that TDP43 binds to its own RNA [45], confirming the reliability of our data. Additionally, TDP43 strongly binds to RNAs, including *Ighv1-60*, which is related to immunity; zinc finger protein 345 (*Zfp345*), which is predicted to enable DNA-binding transcription factor activity; and *Ddx4*, which is closely related to RNA metabolism and development (Fig. 8F). Gene ontology analysis indicated that TDP43-binding RNAs were primarily related to important biological functions, such as transcription activation, DNA damage repair, and protein transport (Fig. 8G). NS-associated transcripts were identified in 293T cells using APEX2-mediated proximity labeling. A total of 2767 human NS-associated transcripts were surveyed by collecting RNAs pulled down by three important NS proteins: SRSF1, SRSF7, and RNPS1 [46] (Fig. 8H). In total, 842 NS-associated transcripts were common to both humans and mice, of which 77 were detected as TDP43-binding RNAs in mouse embryos by RIP-seq (Fig. 8I). To some extent, the RNAs bound by TDP43 correlate with NS-related RNAs. To exclude transcript differences caused by different cell species and cell stages, we overlapped the TDP43-binding RNAs in humans with the NS-associated RNAs shared by these two databases [33, 46] and observed that 45.54% of the NS transcripts were TDP43-binding transcripts (Fig. 8J). However, NS-associated RNAs exhibited minimal overlap with RNAs associated with the nucleolus, nuclear lamina, nuclear pore, ER membrane cytosol-facing (ERM), and outer mitochondrial membrane, with the highest overlap being only 7.04% [46, 47] (Fig. 8J), indicating that the high correlation between TDP43-RNAs and NS-associated RNAs is biologically specific and not coincidental. Additionally, TDP43 deficiency did not disrupt the expression of the TDP43-bound NS-associated RNAs (Fig. 8K), suggesting that TDP43 regulates NS not by affecting RNA quantity, but through other pathways. Based on our findings, we hypothesize that TDP43 generates an active condensation-deficient state after LLPS, which potentially drives NS-RNA and recruits NS-proteins, thereby mediating the assembly of the NS–RNA–protein complex (Fig. 8L).

## Discussion

MZT is an important period for embryonic development. Many studies have demonstrated that transcriptional dysregulation during this period can lead to early embryonic arrest; however, several gaps in our understanding of the mechanisms of post-transcriptional regulation remain. Recent studies have shown that regulation of splicing plays an important physiological role in cell fate conversion and embryonic development. Treatment with the splicing inhibitor PlaB transforms embryonic stem cells into totipotent blastomere-like cells. One study indicated that this was due to the inability of pluripotent genes to be properly spliced, whereas totipotent genes with short introns could be spliced to function [44]. From the perspective of the critical region for splicing (i.e. NS), it has been reported that the loss of important NS components such as SRRM2 and SON leads to NS defects and splicing disturbances [48–50], revealing the importance of NS-mediated splicing regulation. However, these studies were limited to NS-related components, and the key upstream factors that regulate NS during the embryonic period remain unclear.

Biomolecular condensates, which are membraneless cellular compartments formed by LLPS, play an important role in various biological processes, such as mitochondrial regulation, RNA processing, and tumorigenesis [51–53]. However, information on whether LLPS affects the reproductive developmental processes in the germ cells of female mice remains limited. In this study, we demonstrated that TDP43 exhibits LLPS properties in female germ cells. Experimental evidence supporting this view is as follows: (i) droplet-like TDP43 forms in the oocytes; (ii) droplet-like TDP43 underwent reversible division and fusion; (iii) droplet-like TDP43 and diffuse TDP43 can be dynamically exchanged in response to changes in cellular status; and (iv) TDP43 phase-change mutants (TDP43^G335A^; TDP43^Δ316–346^) had a phase-change-disrupted phenotype in 2-cell embryos.

TDP43 and NSs exist in interphase somatic cells, prophase oocytes, and early embryonic cells, except at the cleavage stage. Moreover, TDP43 and NSs have a close and mutually exclusive localization and are conserved across multiple cell types. We found that TDP43 specifically entered the nucleus during the 2-cell period, which is consistent with previous reports [34]. Interestingly, NSs were heavily remodeled during the 2-cell period, when diffuse TDP43 was much higher than during other periods. TDP43, which is localized adjacent to NSs, is indispensable for NS assembly regulation. We found that the condensation-deficient state of TDP43 is the active form that exerts this function. (i) Live cell experiments showed that NS was generated gradually as droplet-like TDP43 transformed into a diffuse state. (ii) The supplementation of TDP43^Δ316–346^ rather than TDP43^G335A^ in *Tdp43^♀–/♂+^* embryos could replenish the formation of NSs. (iii) Overexpression of TDP43^Δ316–346^ increased the number of NSs. (iv) Overexpression of TDP43^G335A^ state TDP43 reduced NS number.

Maternal TDP43 is expressed in oocytes, and zygotic TDP43 is re-expressed after fertilization. Knocking out TDP43 in oocytes (*Tdp43^fl/fl^; Gdf9-Cre* mice) would cause 2-cell arrest. Therefore, we hypothesized that maternal TDP43 plays an important role in 2-cell embryos. In addition, the condensation-deficient state of TDP43 in the 2-cell stage was much higher than that in the other stages, indirectly proving that it can function. Knocking out TDP43 during the zygotic period leads to embryonic arrest in the blastocyst period [54], indicating that TDP43 expression during the embryonic period is also important for embryonic development. However, it is worth noting that maternal TDP43 is the previous threshold for ensuring development. Regarding the lower expression of TDP43 in 2-cell embryos, we believe that low expression and high sensitivity are not contradictory. A change in its localization is important for TDP43 to function.

The NS is a ribonucleoprotein complex. Both RNAs and NS proteins are essential for NS assembly and formation. Treatment of oocytes with the transcription inhibitor α-amanitin would lead to defects in NS assembly [21]. Recent studies have reported that nascent RNAs recruit splice-complex components, supporting this hypothesis [55]. However, TDP43 deficiency does not affect the formation of transcription-related bodies or RNA polymerase II phosphorylation, indicating that TDP43 does not affect NS in a transcription-dependent manner. Additionally, there was no difference in the EU staining results between the knockout and control groups, indicating that TDP43 barely affected nascent transcription. Due to the precious nature of mouse oocyte and zygote samples, coupled with the technical challenges of using these systems, certain mechanistic investigations were infeasible in our study. As a result, we lack direct evidence clarifying how TDP43 facilitates NS assembly. Nevertheless, our analysis revealed that TDP43 can bind to NS proteins. TDP43-binding RNAs were highly correlated with NS-associated RNAs. We propose that these interactions are unlikely coincidental and may reflect TDP43’s essential role in NS organization. Therefore, we propose the following mechanistic hypothesis: TDP43 may serve as a molecular matchmaker between NS-associated RNAs and proteins, concurrently binding RNAs and interacting with NS proteins to enable assembly of the NS complex. This hypothesis necessitates further validation through molecular biology approaches and advanced sequencing technologies. With ongoing developments in low-input high-throughput sequencing methodologies, future studies hold promise for elucidating TDP43-mediated mechanisms governing NS formation and function.

As another nuclear body, the paraspeckle in somatic cells is composed of NONO, SFPQ, PSPC1, and FUS in the core; TDP43 in the shell; and RBM14 and BRG1 in the patch [56]. Recent studies have demonstrated that TDP43 is a core component of paraspeckles in mouse embryos [57]. Deficiency of the core components of the paraspeckle, *Neat1* lncRNA, and NONO causes embryo arrest at the 16- to 32-cell stages. TDP43 deficiency also causes aberrant embryonic development [34, 58, 59]. However, the paraspeckle-deficient phenotype was weaker than that of TDP43 deficiency. Therefore, we speculated that TDP43 is an upstream factor that regulates NSs, in addition to paraspeckles. The droplet-like TDP43 can serve as a storage state for subsequent use. However, whether TDP43 is required for the paraspeckle function remains unclear. Moreover, whether there is a biological exchange or functional assistance between these membraneless organelles remains unclear.

TDP43 deficiency affected NSs in 2-cell embryos but not in oocytes, indicating that TDP43 specifically regulates newly formed NSs during the ZSA process, rather than pre-existing NSs. Furthermore, the regulatory effect of TDP43 on NSs was correlated with the TDP43 dosage. When TDP43 was deficient, NSs could not form normally in 2-cell embryos, whereas the surge in the condensation-deficient state of TDP43 led to larger and more NSs within the embryos. TDP43 primarily regulates skipping events during pre-mRNA processing. NS defects caused by disturbances in TDP43 dosage also led to splicing disorders. Specifically, TDP43 deficiency prevents normal exon skipping, whereas excessive TDP43 expression increases exon skipping events. Abnormal splicing can be fatal during embryonic development. Based on these results, we propose a hypothesis for NS operations. Because DNA transcription produces many transcripts, TDP43 acts as a matchmaker, binding to these RNAs and NS-associated proteins and promoting the construction of NSs. Subsequently, splicing and RNA processing factors begin to function in the NSs. Nascent mRNAs were spliced, tailed, and ultimately matured to ensure normal biological function (Supplementary Fig. S10F).

We noticed that embryonic arrest caused by TDP43 dysregulation almost always occurred in the 2-cell stage, which is a critical period for the transition from maternal-to-zygotic control. It is also a node for the transition from the early embryonic stage to pluripotency. Therefore, we examined the effect of TDP43 deficiency on the transition from totipotency to pluripotency and found that defects in ZSA mediated by abnormal TDP43 levels also caused abnormal cell fate determination. This abnormal conversion of totipotency to pluripotency was similar to the effect of PlaB splicing inhibitor treatment, providing further evidence for the physiological significance of alternative splicing in embryos.

In conclusion, we have identified a key upstream regulatory factor of NS, TDP43, which is essential for ZSA in mouse embryos. The mechanism underlying this is important for understanding the splicing regulatory network and reflects the multiple functions of TDP43 in regulating RNA metabolism.

## Supplementary Material

gkaf1469_Supplemental_Files

## Data Availability

Poly(A) RNA-seq data from the embryos were deposited in the NCBI Gene Expression Omnibus database under the accession code GSE278472. RIP-seq data from the embryos were deposited in the NCBI Gene Expression Omnibus database under the accession code GSE278470.
