# Supplementary material for: Maternal TDP43 orchestrates nuclear speckle assembly and zygotic splicing activation during oocyte-to-embryo transition in mice
Source: Nucleic Acids Res. 2026 Jan 9;54(1):gkaf1469. doi: 10.1093/nar/gkaf1469 (PMC12784972; doi:10.1093/nar/gkaf1469)
Supplement: gkaf1469_Supplemental_Files [file gkaf1469_supplemental_files.zip › 12032025-TDP43-supplement.pdf]

Supplementary Materials

Supplementary Figures

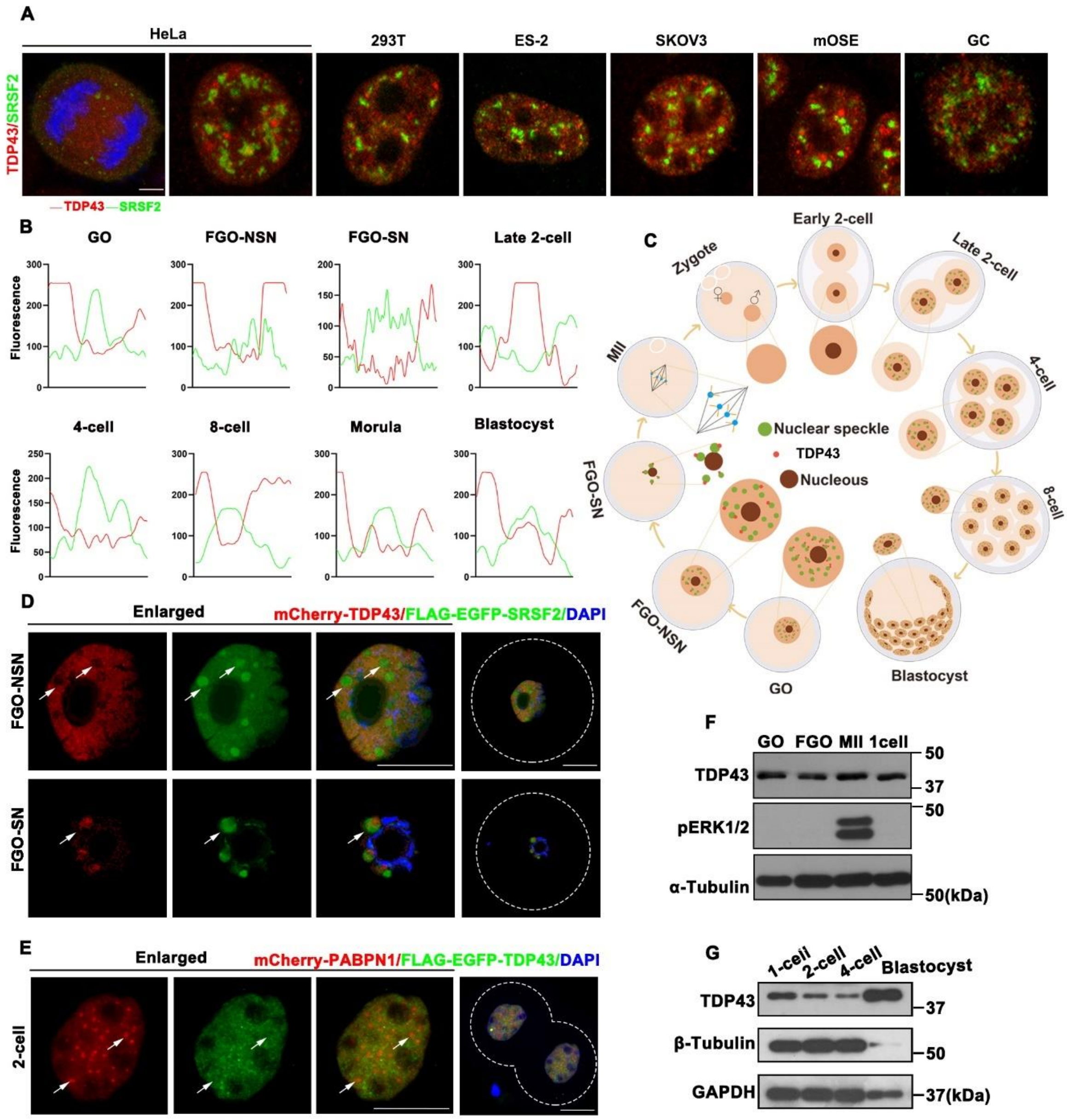

Figure S1. Localization analysis between TDP43 and NSs in human somatic cells, mouse oocytes and 2-cells. A: Representative images indicating the position relationship between TDP43 and SRSF2 in HeLa, 293T, ES-2, SKOV3, mOSE cells, and mouse granulosa cells (GCs). Scale bar, 5 μm. **B:** Plot profile analyses of the relative fluorescence intensity of signals indicated by the white dashes in Fig. 1F. **C:** Schematic diagram represents the position changes from Fig. 1F. **D-E:** Immunofluorescence images indicating the expression and localization of exogenously injected TDP43 and SRSF2 in oocytes and 2-cells. Scale bar, 20 μm. **F-G:** Western

blotting results of TDP43 levels from growing oocytes (GOs) to blastocysts. Total proteins from 200 oocytes or embryos were loaded into each lane. α-Tubulin and GAPDH were loaded as loading controls.

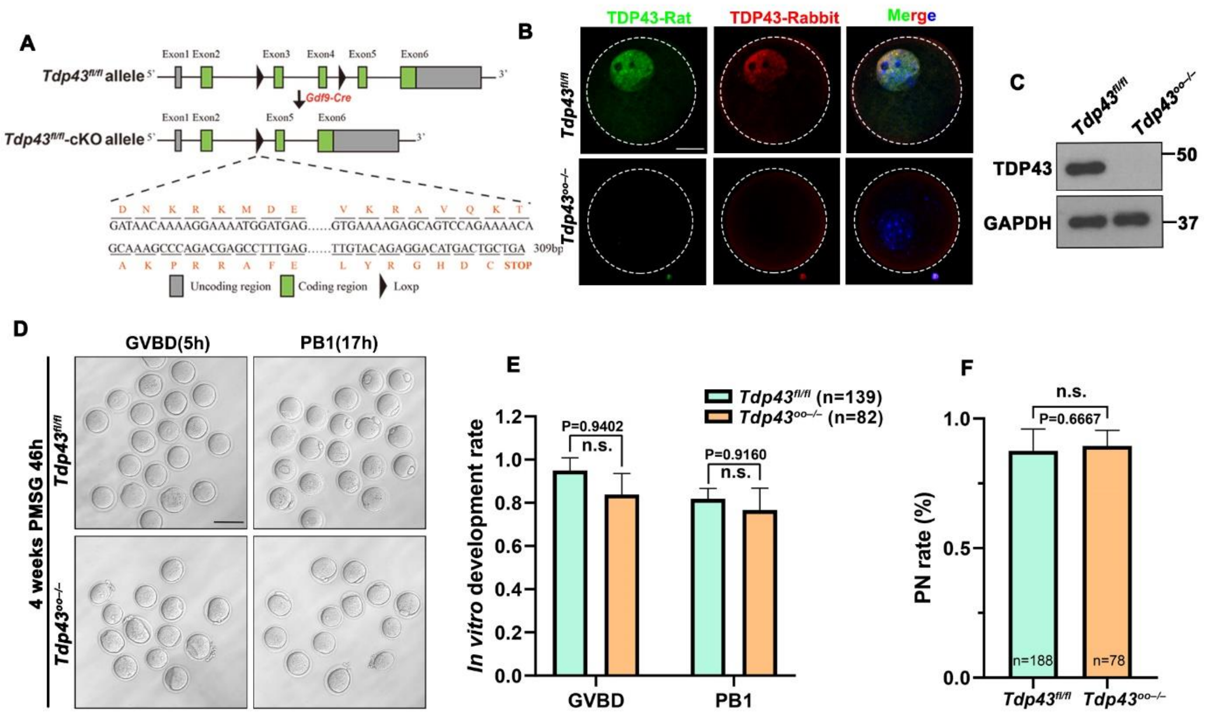

Figure S2. *Tdp43*-depletion did not affect oocyte maturation. A: The diagram indicating the acquisition of *Tdp43* conditional knockout mice hybridized with *Gdf9-Cre* mice. **B:** Immunofluorescence results indicating the knockout efficiency of TDP43 used with rabbit and rat anti-TDP43 antibodies, respectively. Scale bar, 20 μm. **C:** Western blots indicating the knockout efficiency of TDP43. Total proteins from 150 oocytes were loaded into each lane. GAPDH was loaded as a loading control. **D:** Representative DIC images of GVBD oocytes and PB1 emitted oocytes cultured *in vitro* from 4-week-old control and knockout mice. Scale bar, 100 μm. **E:** Rates of GVBD and PB1 emission in oocytes from (D). Data are presented as mean ± SEM. n.s. indicates non-significant. The number of analyzed oocytes was indicated (n). **F:** Analysis of pronucleus (PN) formation in $Tdp43^{fl/fl}$ and $Tdp43^{oo-/-}$ oocytes. Data are presented as mean ± SEM. n.s. indicates non-significant. The number of analyzed fertilized eggs is indicated (n).

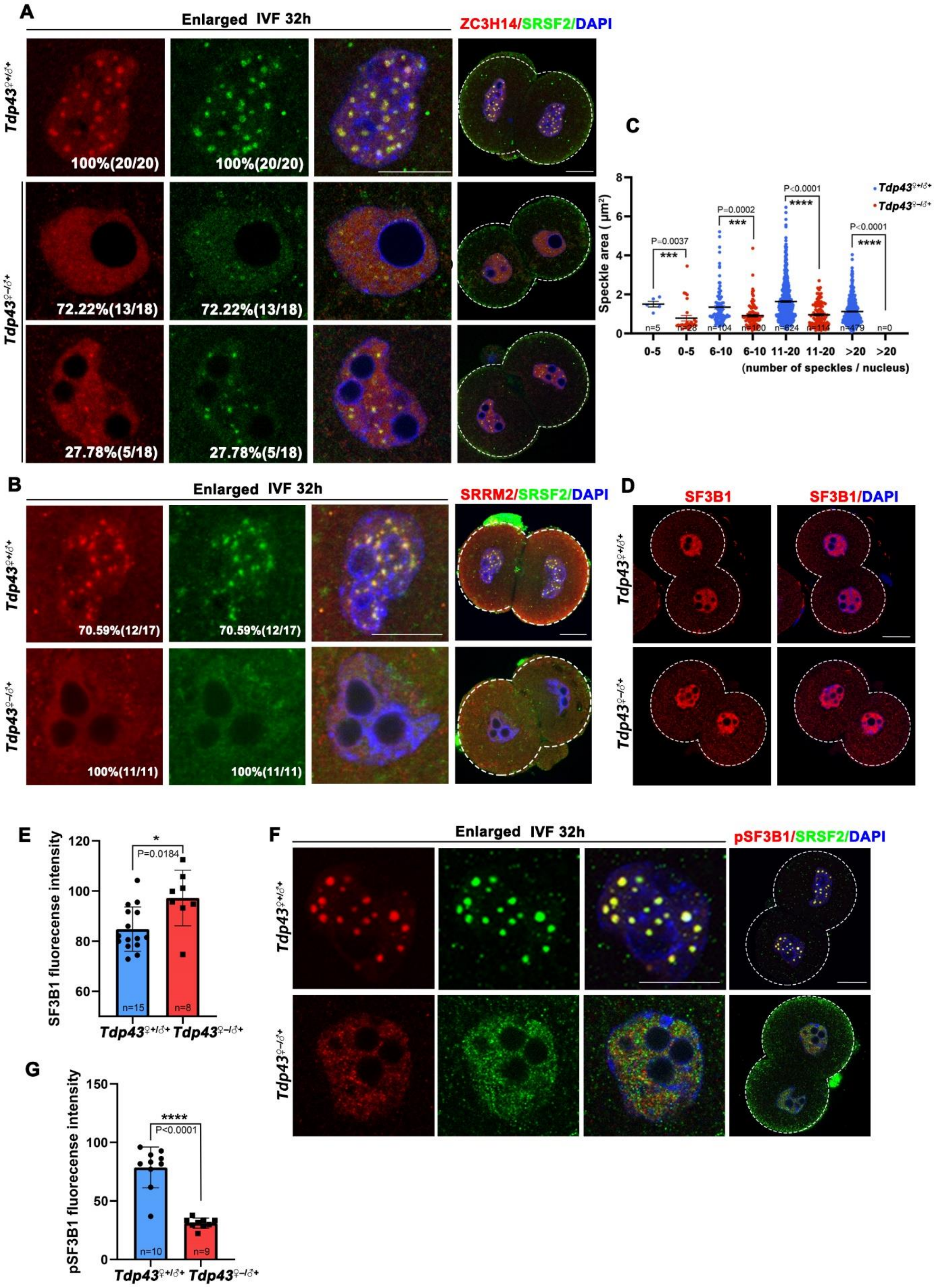
A
Enlarged IVF 32h
ZC3H14/SRSF2/DAPI
Tdp43♀+/♂+
Tdp43♀−/♂+
100%(20/20)
100%(20/20)
72.22%(13/18)
72.22%(13/18)
27.78%(5/18)
27.78%(5/18)
B
Enlarged IVF 32h
SRRM2/SRSF2/DAPI
70.59%(12/17)
70.59%(12/17)
100%(11/11)
100%(11/11)
C
Speckle area (μm²)
P=0.0037
P=0.0002
P<0.0001
P<0.0001
n=5
n=28
n=104
n=100
n=624
n=114
n=479
n=0
0-5
6-10
11-20
>20
(number of speckles / nucleus)
D
SF3B1
SF3B1/DAPI
E
SF3B1 fluorecense intensity
P=0.0184
n=15
n=8
F
Enlarged IVF 32h
pSF3B1/SRSF2/DAPI
G
pSF3B1 fluorecense intensity
P<0.0001
n=10
n=9

27

Figure S3. Deficiency of maternal TDP43 disrupted NS assembly in 2-cells. A: Immunofluorescence images providing the distribution of NS-associated proteins (ZC3H14 and SRSF2) in 2-cells at IVF 32h of control and *Tdp43*-knockout females. Scale bar, 20 µm. **B:** Immunofluorescence images providing the distribution of NS-associated proteins (SRRM2 and SRSF2) in 2-cells at IVF 32h of control and *Tdp43*-knockout females. Scale bar, 20 µm. **C:** Quantitative statistics indicating the NS areas in $Tdp43^{♀+/♂+}$ and $Tdp43^{♀-/♂+}$ 2-cells according to the NS density per blastomere. The number of analyzed speckles is indicated (n). Error bars, S.E.M. *** P < 0.001; **** P < 0.0001 by two-tailed Student's t-test. **D:** Immunofluorescence indicating the expression and distribution of SF3B1 in 2-cells. Scale bar, 20 µm. **E:** Quantification of SF3B1 intensity signal of (D). The number of analyzed 2-cells is indicated (n). Error bars, S.E.M. * P < 0.05 by two-tailed Student's t-test. **F:** Immunofluorescence indicating the localization of phosphorylated SF3B1 within NSs of 2-cells. Scale bar, 20 µm. **G:** Quantification of pSF3B1 intensity signal of (F). The number of analyzed 2-cell is indicated (n). Error bars, S.E.M. **** P < 0.0001 by two-tailed Student's t-test.

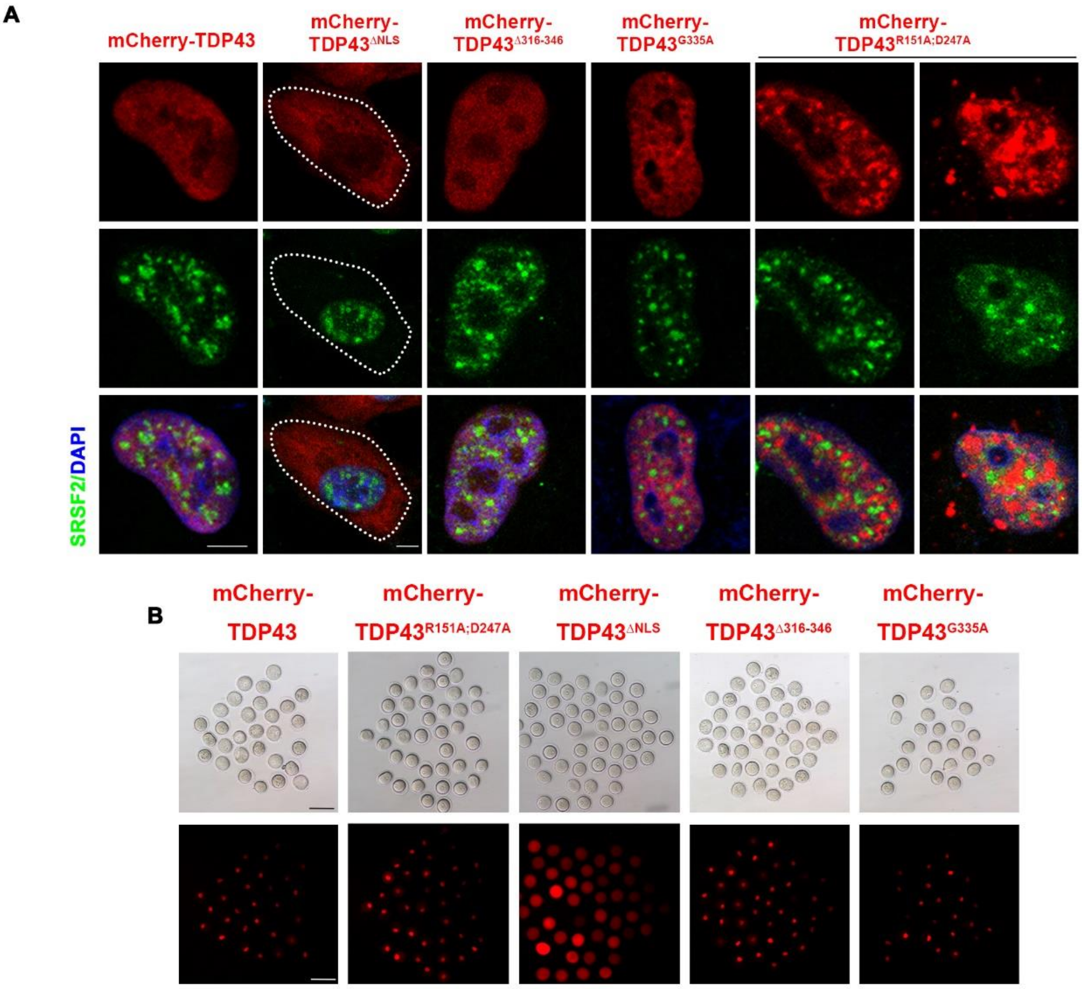

Figure S4. Expression verification of TDP43 and its mutants. A: Immunofluorescence images indicating the expression and localization of TDP43 and its mutants after transfection into 293T cells. The White dashed line indicates the cell outline. Scale bar, 2.5 μm. **B:** Representative images of FGOs injected with *Tdp43* and its mutant mRNA, validating the quality of *in vitro* transcribed mRNA. Scale bar, 100 μm.

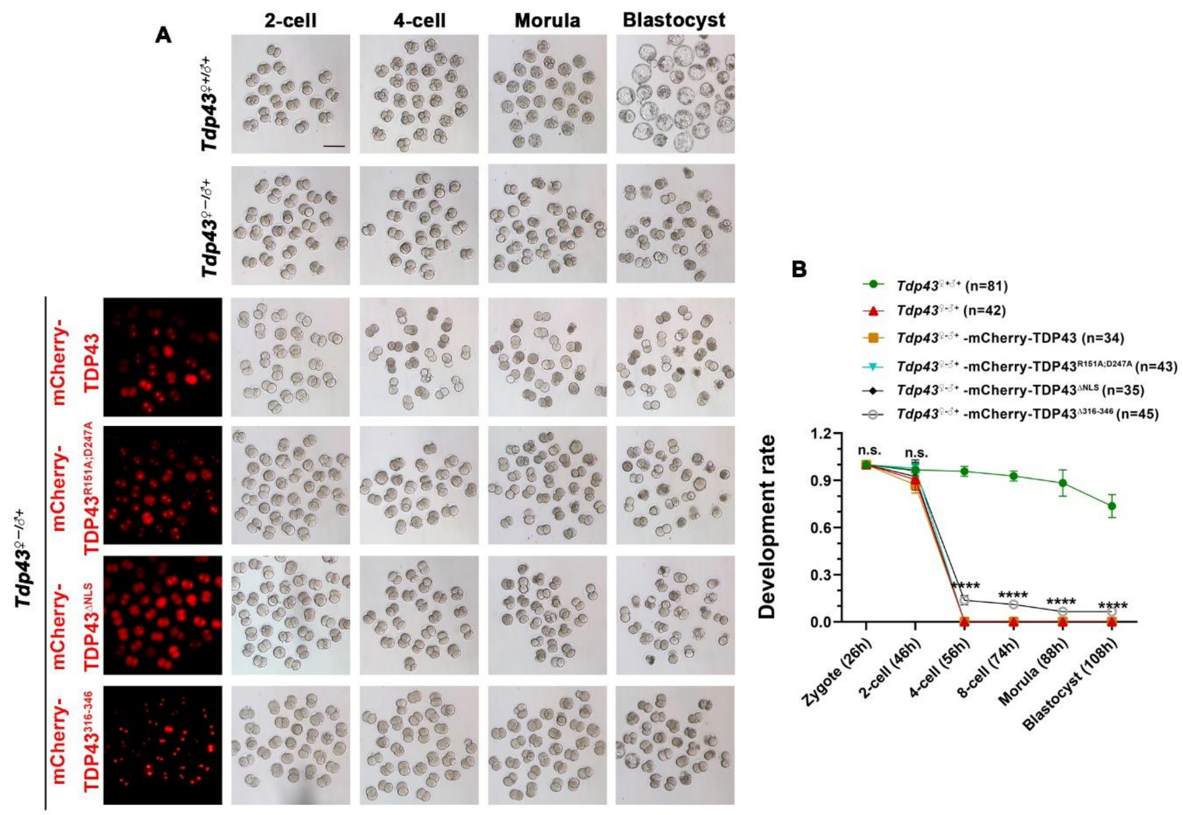

Figure S5. Zygotically supplementation of TDP43 could not rescue the development failure of maternal TDP43-deficient embryos. A: Representative images indicating the development of embryos after injection of *Tdp43* and its mutant mRNA in *Tdp43*$^{♀-/♂+}$ zygotes. **B:** Statistics of embryo development rates from (A). The number of analyzed embryos is indicated (n). Error bars, S.E.M. ****P < 0.0001 by two-tailed Student's t-test, n.s. indicates non-significant.

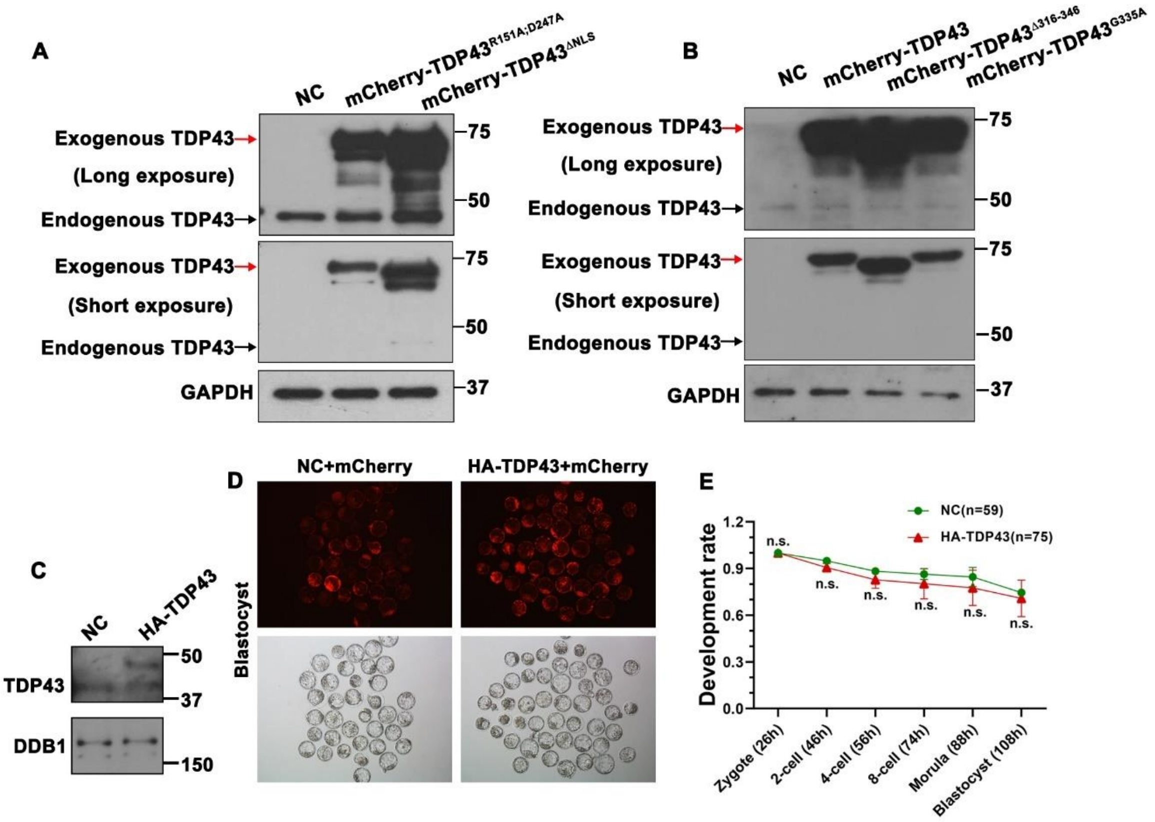

Figure S6. Exogenous TDP43 is much greater than endogenous TDP43 to cause embryonic arrest. A-B: Western blots indicating the expression level of *Tdp43* and its mutant mRNAs transcribed *in vitro*. Total proteins from 80 2-cells were loaded in each lane. GAPDH served as the loading control. **C:** Western blots showing the injection volume was adjusted to make the expression levels of exogenous TDP43 and endogenous TDP43 consistent. DDB1 served as the loading control. Total proteins from 80 2-cells were loaded in each lane. **D-E:** Analysis of the effect on embryonic development when exogenous TDP43 with the same expression level as endogenous TDP43 is injected into zygotes. The number of analyzed embryos is indicated (n). Error bars, S.E.M. n.s. indicates non-significant.

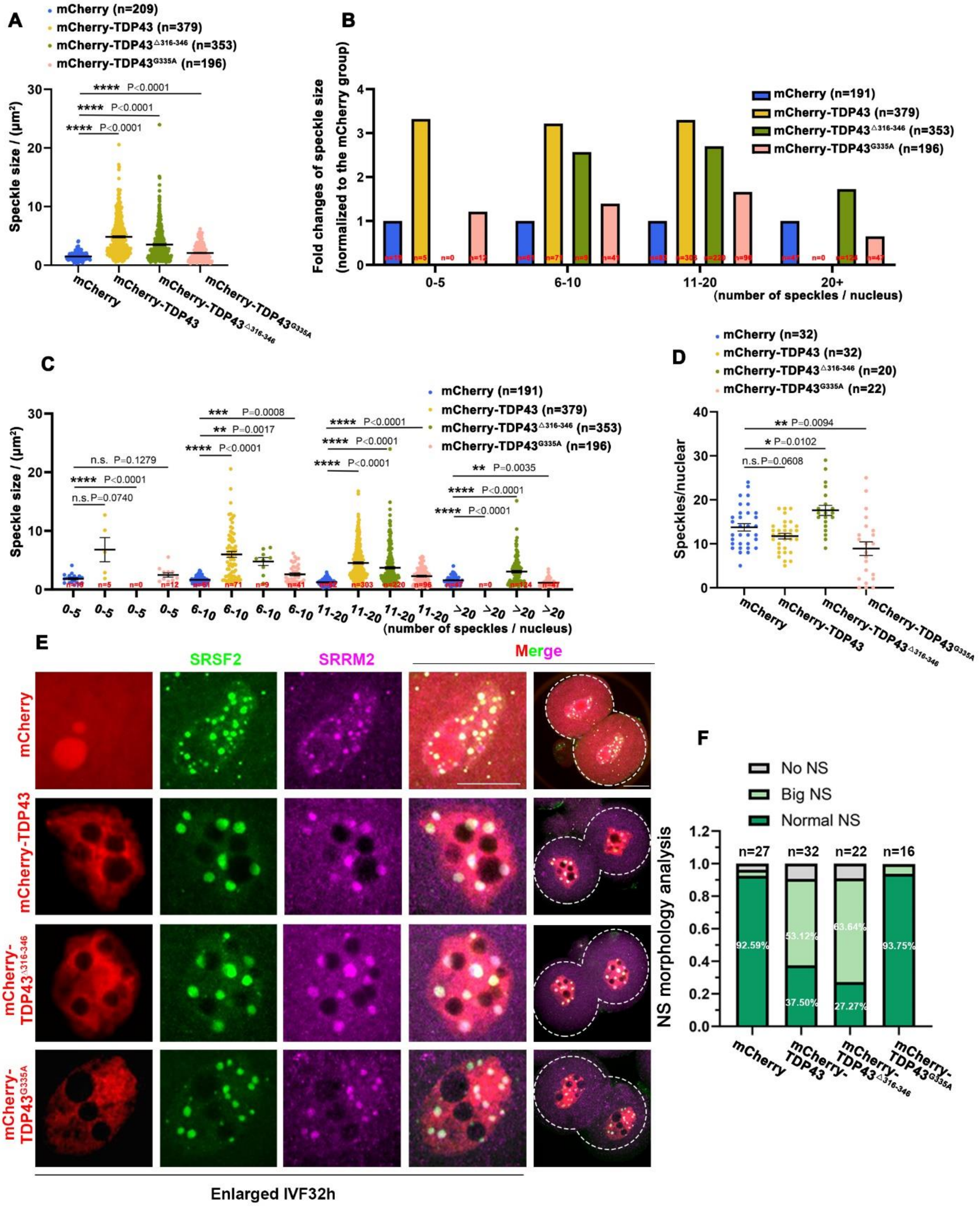

Figure S7. Statistical analysis of NS in 2-cell embryos after the injection of *Tdp43* and its mutants into zygotes. A: Statistical data indicating the areas of NSs in 2-cells. Error bars, S.E.M. **** P < 0.0001 via two-tailed Student's t-test. The number of analyzed speckles is indicated (n). **B-C:** Quantitation of the NS areas in the control, TDP43, and TDP43 variants overexpressed in 2-cells according to the NS amounts per blastomere. Error bars, S.E.M. ** P

< 0.01; **** P < 0.0001 via two-tailed Student's t-test. n.s. indicates non-significant. The number of analyzed speckles was indicated (n). **D:** Statistical data indicating the number of NSs in the indicated 2-cells. Error bars, S.E.M. * P < 0.05; ** P < 0.01 via two-tailed Student's t-test. n.s. means non-significant. The number of analyzed speckles is indicated (n). **E:** Immunofluorescence images indicating the status of NS-associated proteins (SRRM2 and SRSF2) in 2-cell after injection TDP43 and its mutants in the zygote. Scale bar = 20 μm. **F:** Statistics of NSs existence in 2-cell embryos from (E). The number of analyzed 2-cell embryos is indicated (n).

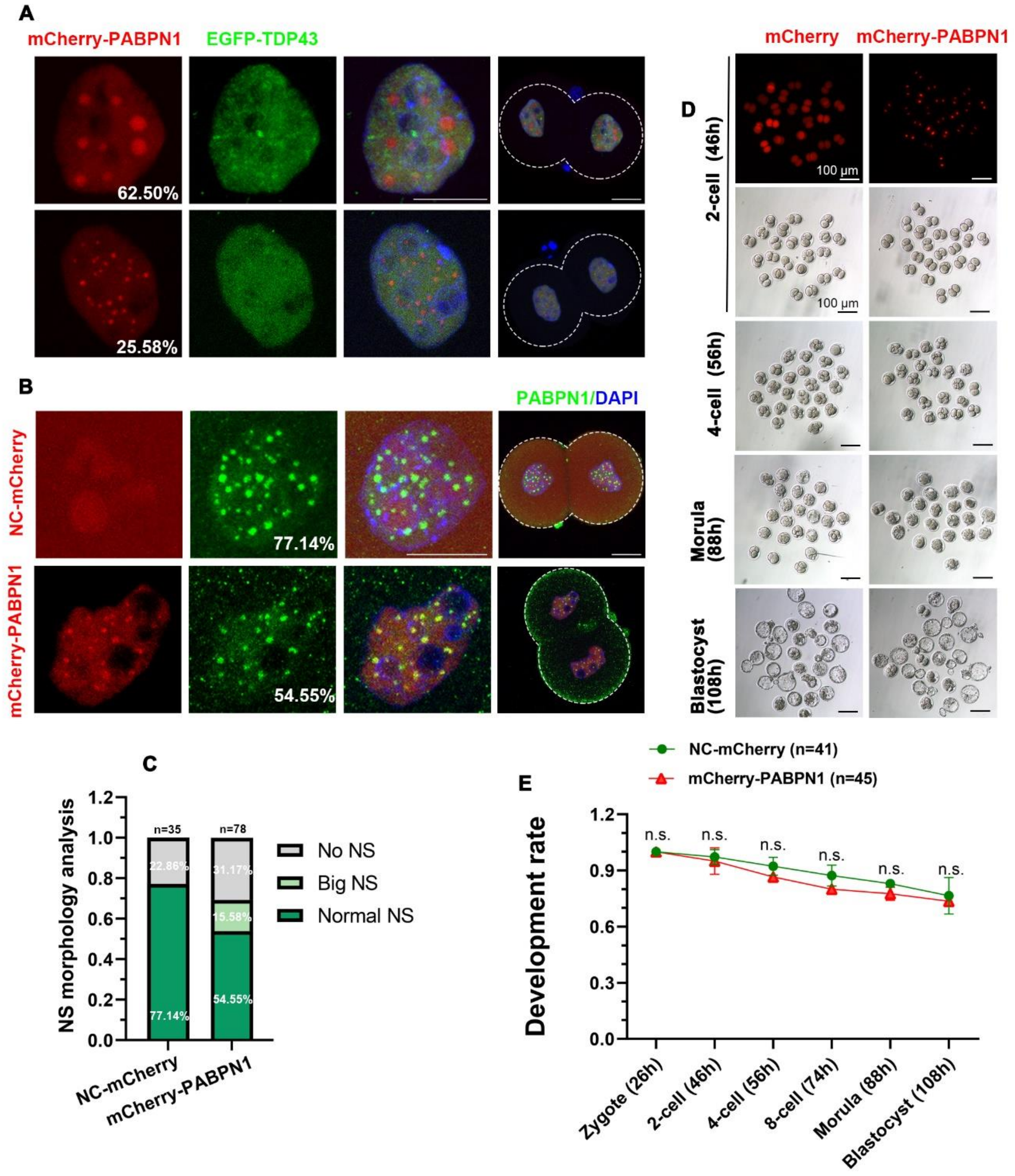

Figure S8. Excessive PABPN1 did not interfere with NS or embryonic development. A: Representative images indicating the expression and localization of exogenously expressed mCherry-PABPN1 and EGFP-TDP43. Scale bar, 20 μm. **B:** Immunofluorescence staining images indicating the state of the NS after injection of *mCherry* or *mCherry-Pabpn1*. Scale bar, 20 μm. **C:** Statistics detailing NS existence in 2-cells from (B). The number of analyzed 2-cells is indicated (n). **D:** Representative images of embryos injected with *mCherry* or *mCherry-Pabpn1* mRNAs at various developmental stages. Fluorescence images indicating the translation efficiency of *in vitro*-transcribed mRNAs. All embryos were microinjected at 6-8 hours after fertilization. Scale bar, 100 μm. **E:** Statistics of the embryo development rate from (D). The number of embryos analyzed is presented (n). Error bars, S.E.M., n.s. indicates not significant.

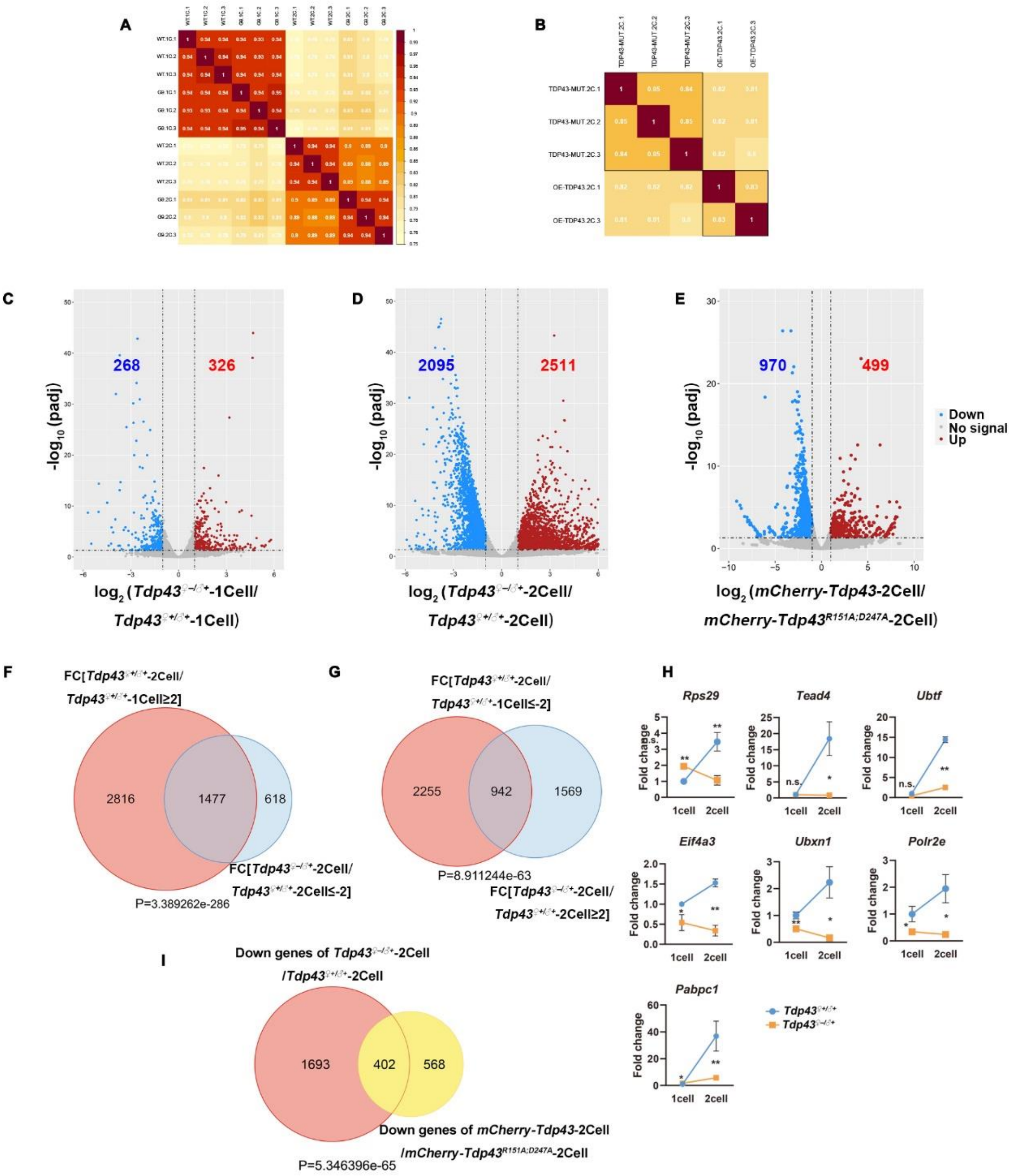

Figure S9. Transcriptome analysis of wild-type, TDP43-deficient, and TDP43-overexpressing zygotes and 2-cells. A-B: Heatmaps indicating spearman correlation coefficients of normal/TDP43-deficient group (A) and $TDP43^{R151A;D247A}$-overexressing/TDP43-overexpressing group (B). **C-E:** Volcano plots providing the number of significantly differentially expressed genes (DEGs) in zygotes and 2-cells from wild-type (WT)/TDP43-deficient (C-D) and $TDP43^{R151A;\ D247A}$-overexpressing/TDP43-overexpressing (E) embryos. Numbers of up-regulated and down-regulated transcripts are indicated in red and blue, respectively. **F:** Venn diagram indicating the overlap of transcripts up-regulated during WT

zygote-to-2-cell transition and transcripts down-regulated in TDP43-deficient 2-cell embryos compared to WT controls. **G:** Venn diagram indicating the overlap of transcripts down-regulated during WT zygote-to-2-cell transition and transcripts up-regulated in TDP43-deficient 2-cell embryos compared to WT controls. **H:** RT-qPCR results indicating the changes of transcripts from zygote to 2-cell in WT and TDP43-deficient embryos. Data are presented as mean ± SEM. * $P < 0.05$; ** $P < 0.01$ by two-tailed Student's t-test. n.s. indicates non-significant. **I:** Venn diagram indicating the overlap of down-regulated transcripts in 2-cells between WT/TDP43-deficient group and the TDP43$^{R151A;D247A}$-overexpressing/TDP43-overexpressing group.

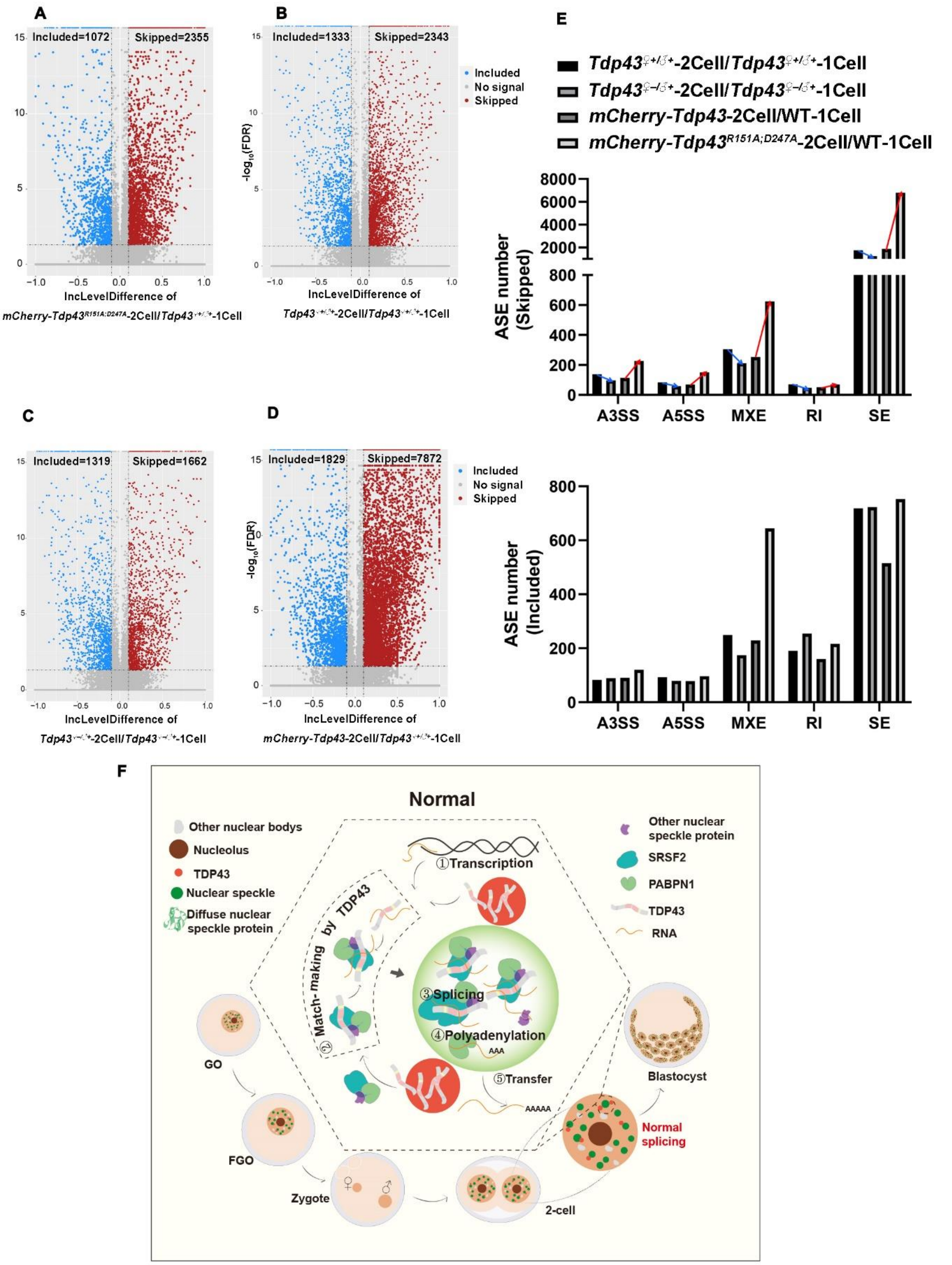

Figure S10. Alternative splicing events in TDP43-deficient, and TDP43-excessive embryos, corresponding to Figure 6. A-B: Volcano plots indicating the number of significantly differentially ASEs between TDP43$^{R151A;\ D247A}$-overexpressing (A) and WT (B) embryos from

zygotes to 2-cells. The numbers of skipped and included events are indicated in red and blue. **C-D:** Volcano plots indicating the number of significantly differentially alternative splicing events (ASEs) between TDP43-deficient (C) and TDP43-overexpressing (D) embryos from zygotes to 2-cells. The numbers of skipped and included events are indicated in red and blue, respectively. **E:** The numbers of skipped events and included events of A3SS, A5SS, MXE, and RI during ZGA. The blue arrow indicates a downward trend, and the red arrow indicates an uptrend. All analysis of ASEs in Figure S10 was defined as FDR < 0.05 and |ILD| > 0.1. FDR, false discovery rate; ILD, Inclusion Level Difference. **F:** A proposed model of TDP43 regulating NS assembly and its physiological function in embryonic development.

Supplementary Tables

Table S1. Antibody information.

**Protein/Target name**	**Manufacture (catalogue number)**	**Application (working dilution)**	**Website link**
FLAG	Sigma (F3165)	WB (1:3000)	http://www.sigmaaldrich.com/catalog/product/sigma/f3165?lang=zh&region=CN
HA	Cell Signaling Technology (3724)	WB (1:1000)	https://www.cellsignal.com/products/primary-antibodies/ha-tag-c29f4-rabbit-mab/3724
MYC	Invitrogen (13-2500)	WB (1:2000)	https://www.thermofisher.cn/cn/zh/antibody/product/c-Myc-Antibody-clone-9E10-Monoclonal/13-2500
IgG	Cell Signaling Technology (3900)	endo IP	https://www.cst-c.com.cn/products/primary-antibodies/rabbit-da1e-mab-igg-xp-isotype-control/3900?site-search-type=Products
TDP43	ProteinTech (10782)	WB (1:1000)<br>IF (1:400)<br>endo IP	https://www.ptglab.com/products/TARDBP-Antibody-10782-2-AP.htm
	Biolegend (808301)	IF (1:200)	https://www.biolegend.com/en-us/products/purified-anti-tdp43-antibody-11572
SRSF2	Sigma-Aldrich (SAB4200725)	IF (1:100)<br>WB (1:1000)	https://www.sigmaaldrich.cn/CN/zh/product/sigma/sab4200725
	ABclonal (A3635)	WB (1:500)	https://abclonal.com.cn/catalog/A3635
PABPN1	Bethy (A303-523A)	WB (1:1000)<br>IF (1:200)	https://www.thermofisher.cn/cn/zh/antibody/product/PABPN1-Antibody-Polyclonal/A303-523A
ZC3H14	ProteinTech (28190)	WB (1:1000)<br>IF (1:200)	https://www.ptglab.co.jp/products/ZC3H14-Antibody-28190-1-AP.htm

pSF3B1	Affinity (AF2401)	IF (1:200)<br>WB (1:1000)	https://www.affbiotech.com/goods-15700-AF2 401-Phospho_SF3B1_Thr313_Antibody.html
SF3B1	ABclonal (A15801)	IF (1:200)	https://abclonal.com.cn/catalog/A15801
RNA PolII (pS2)	Abcam (ab5095)	IF (1:40000)	http://www.abcam.cn/rna-polymerase-ii-ctd-rep eat-ysptsps-phospho-s2-antibody-chip-grade-ab 5095.html
MuERVL - Gag	Huabio (ER50102)	IF (1:200)	https://www.huabio.com/products/muervl-gag antibody-polyclonal-er50102
γH2AX	Cell Signaling (#9718S)	IF (1:400)	https://www.cellsignal.com/products/primary-antibodies/phospho-histone-h2a-x-ser139-20e3-rabbit-mab/9718
GAPDH	Trans (HC301)	WB (1:1000)	https://www.transgenbiotech.com/loading_cont rols/proteinfind_anti_gapdh_mouse_monoclon al_antibody.html
α-Tubulin	Cell Signaling Technology (2144)	WB (1:1000)	https://www.cellsignal.com/products/primary-antibodies/a-tubulin-antibody/2144
pERK1/2	Cell Signaling (9101)	WB (1:1000)	https://www.cellsignal.com/products/primary-antibodies/phospho-p44-42-mapk-erk1-2-thr202-tyr204-antibody/9101
SRRM2	ProteinTech (30741)	IF (1:100)	https://ptglab.co.jp/products/SRRM2-Antibody-30741-1-AP.htm

Table S2. Primer sequences.

**Primer name**	**Genes targeted**	**Application**	**Sequences (5′-3′)**
m*Mervl*-F	mouse *Mervl*	Real-time PCR	ATCGAAAGGCTCCAGACACAA
m*Mervl*-R		Real-time PCR	TTCAGCCAACCTTACAATGAGAG
m*Zscan4b*-F	mouse *Zscan4b*	Real-time PCR	AGCAAAAGACCTTCAGACCAAC
m*Zscan4b*-R		Real-time PCR	GGGGAAAAGTTTAGCTGAGCAC
m*Zscan4c*-F	mouse *Zscan4c*	Real-time PCR	GCCTTATGTCTGTTCCCTATGT
m*Zscan4c*-R		Real-time PCR	CAGTCTCTGCTGAGGATGTTAG
m*Zscan4d*-F	mouse *Zscan4d*	Real-time PCR	GCAGATGCCAGTAGACACCA
m*Zscan4d*-R		Real-time PCR	GGCATCAAGAGGGAATTGAA
m*Tfrc*-F	mouse *Tfrc*	Real-time PCR	ATGCCGACAATAACATGAAGGC

m*Tfrc*-R		Real-time PCR	ACACGCTTACAATAGCCCAGG
m*Klf2*-F	mouse *Klf2*	Real-time PCR	CTCAGCGAGCCTATCTTGCC
m*Klf2*-R		Real-time PCR	CACGTTGTTTAGGTCCTCATCC
m*Piwil2*-F	mouse *Piwil2*	Real-time PCR	TTGGCCTCAAGCTCCTAGAC
m*Piwil2*-R		Real-time PCR	GAACATGGACACCAAACCTACA
m*Dppa4*-F	mouse *Dppa4*	Real-time PCR	AGTCAACCTAGCACGGCTC
m*Dppa4*-R		Real-time PCR	TCCTGGCGTCTCAGTGTCT
m*Rps29*-F	mouse *Rps29*	Real-time PCR	GTCTGATCCGCAAATACGGG
m*Rps29*-R		Real-time PCR	AGCCTATGTCCTTCGCGTACT
m*Tead4*-F	mouse *Tead4*	Real-time PCR	CAACCTGGAACATCCCACGAT
m*Tead4*-R		Real-time PCR	GAAAGCCGAGAACTCCAACAT
m*Ubtf*-F	mouse *Ubtf*	Real-time PCR	CCGCTGGTCCCAGGAAGATA
m*Ubtf*-R		Real-time PCR	CGACTCTGTGGTTTTGAACTTGG
m*Eif4a3*-F	mouse *Eif4a3*	Real-time PCR	AGGAGGACATGACCAAAGTGG
m*Eif4a3*-R		Real-time PCR	TGCTGAATCGCTGAAGGTTTTT
m*Ubxn1*-F	mouse *Ubxn1*	Real-time PCR	TCGAGGCTGCGATGGATTG
m*Ubxn1*-R		Real-time PCR	CAGGGCCAACTTGCTCTGAG
m*Polr2e*-F	mouse *Polr2e*	Real-time PCR	GGTGGGCATCAAGACCATCAA
m*Polr2e*-R		Real-time PCR	TCAGGGACTAGCTCGTGCTC
m*Pabpc1*-F	mouse *Pabpc1*	Real-time PCR	CAAGCCAGTACGCATCATGTG
m*Pabpc1*-R		Real-time PCR	TGCTTCCTGTGTTTCAAAGTGT
m*Gapdh*-F	mouse *Gapdh*	Real-time PCR	AGGTCGGTGTGAACGGATTTG
m*Gapdh*-R		Real-time PCR	TGACCTCAACTACATGGTCTACA
m*Tdp43*-F	mouse *Tdp43*	Genotyping	CCAAACTGACCTCAACTGCTCTGC

m*Tdp43*-R		Genotyping	CTCGGGAATTTCAAAGACAGGCTC
Gdf9-Cre-F	*Gdf9-Cre*	Genotyping	GTGCAAGCTGAACAACAGGA
Gdf9-Cre-R		Genotyping	AGGGACACAGCATTGGAGTC
m*Tnfsf13b*-F	mouse *Tnfsf13b*	Splicing events verification	ATGCCGCCATTCTCAACATG
m*Tnfsf13b*-R		Splicing events verification	CTGGCTGTAGATGAAGAAATAGCCTG
m*Med27*-F	mouse *Med27*	Splicing events verification	CCTGGGTACTGCAGTTGACTTTGTGTAA
m*Med27*-R		Splicing events verification	TCTGTCAATGCGGCTGATCACG
m*Hmg20b*-F	mouse *Hmg20b*	Splicing events verification	AGGCCTACAAGGTCTGCACTGA
m*Hmg20b*-R		Splicing events verification	TGGTCCAGAAACTCTTCCGTGA

Table S3. Quality control of RNA-seq and RIP-seq results.

**Sample**	**Total reads**	**Mapping efficiency**	**ERCC percentage**
WT.1C.1	22011094	87.65%	0.77%
WT.1C.2	23157409	86.70%	0.89%
WT.1C.3	19456353	88.79%	0.70%
G9.1C.1	22074306	88.33%	0.79%
G9.1C.2	21719423	86.62%	0.71%
G9.1C.3	21871549	88.18%	0.60%
WT.2C.1	23124570	74.56%	2.2%
WT.2C.2	22980382	76.75%	2.29%
WT.2C.3	22881320	72.25%	1.73%
G9.2C.1	23232482	67.00%	2.41%
G9.2C.2	21693693	69.72%	2.32%
G9.2C.3	21690963	67.19%	1.7%

TDP43-MUT.2C.1	15853854	70.64%	2.62%
TDP43-MUT.2C.2	14855628	71.56%	2.56%
TDP43-MUT.2C.3	14996066	75.14%	2.42%
OE-TDP43.2C.1	15418780	71.24%	3.17%
OE-TDP43.2C.2	15413624	71.98%	2.96%
2C.*Flag*.1	26932433	40.74%	-
2C.*Flag*.2	28469018	41.06%	-
2C.*Flag*.3	28016040	40.59%	-
2C.*Flag-Tdp43*.1	30994964	72.98%	-
2C.*Flag-Tdp43*.2	31283700	72.78%	-
2C.*Flag-Tdp43*.3	28235803	72.75%	-

Table S4. Featurecounts of transcripts deprived from Smart-seq of *Tdp43*$^{♀+/♂+}$ and *Tdp43*$^{♀-/♂+}$ groups (In a separate xlsx file).

Table S5. Featurecounts of transcripts deprived from Smart-seq of 2C.*mCherry-Tdp43* and 2C.*mCherry-Tdp43*$^{R151A;D247A}$ groups (In a separate xlsx file).

Table S6. FPKMs of transcripts deprived from RIP-seq (In a separate xlsx file).
